# Amino-Functionalized Nitrogen-Doped Graphene-Quantum-Dot-Based Nanomaterials with Nitrogen and Amino-Functionalized Group Content Dependence for Highly Efficient Two-Photon Bioimaging

**DOI:** 10.3390/ijms21082939

**Published:** 2020-04-22

**Authors:** Wen-Shuo Kuo, Chia-Yuan Chang, Keng-Shiang Huang, Jui-Chang Liu, Yu-Ting Shao, Chih-Hui Yang, Ping-Ching Wu

**Affiliations:** 1 School of Chemistry and Materials Science, Nanjing University of Information Science and Technology, Nanjing 210044, China; 2 Allergy & Clinical Immunology Research Center, National Cheng Kung University Hospital, College of Medicine, National Cheng Kung University, Tainan 701, Taiwan; awsklvzzy@gmail.com (J.-C.L.); cherry80021517@gmail.com (Y.-T.S.); 3 Department of Mechanical Engineering, National Cheng Kung University, Tainan 701, Taiwan; cychang0829@gs.ncku.edu.tw; 4 The School of Chinese Medicine for Post-Baccalaureate, I-Shou University, Kaohsiung 840, Taiwan; huangks@isu.edu.tw; 5 Department of Biochemistry and Molecular Biology, National Cheng Kung University Hospital, College of Medicine, National Cheng Kung University, Tainan 701, Taiwan; 6 Department of Microbiology & Immunology, National Cheng Kung University Hospital, College of Medicine, National Cheng Kung University, Tainan 701, Taiwan; 7 Department of Biological Science and Technology, I-Shou University, Kaohsiung 840, Taiwan; 8 Pharmacy Department of E-Da Hospital, Kaohsiung 824, Taiwan; 9 Taiwan Instrument Research Institute, National Applied Research Laboratories, Hsinchu City 300, Taiwan; 10 Department of Biomedical Engineering, National Cheng Kung University, Tainan 701, Taiwan

**Keywords:** graphene quantum dot, two-photon photoproperties, ultralow two-photon excitation power, two-photon autofluorescence, noninvasive three-dimensional imaging

## Abstract

We fabricated nanomaterials comprising amino-functionalized and nitrogen-doped graphene quantum dots (amino-N-GQDs) and investigated their photostability and intrinsic luminescence in the near-infrared spectrum to determine their suitability as contrast agents in two-photon imaging (TPI). We observed that amino-N-GQDs with a higher amount of bonded nitrogen and amino-functionalized groups (6.2%) exhibited superior two-photon properties to those with a lower amount of such nitrogen and groups (4.9%). These materials were conjugated with polymers containing sulfur (polystyrene sulfonate, PSS) and nitrogen atoms (polyethylenimine, PEI), forming amino-N-GQD–PSS–PEI specimens (amino-N-GQD-polymers). The polymers exhibited a high quantum yield, remarkable stability, and notable two-photon properties and generated no reactive oxygen species, rendering them excellent two-photon contrast agents for bioimaging. An antiepidermal growth factor receptor (Ab_EGFR_) was used for labeling to increase specificity. Two-photon imaging (TPI) of amino-N-GQD (6.2%)-polymer-Ab_EGFR_-treated A431 cancer cells revealed remarkable brightness, intensity, and signal-to-noise ratios for each observation at a two-photon excitation power of 16.9 nJ pixel^−1^ under 30 scans and a three-dimensional (3D) depth of 105 µm, indicating that amino-N-GQD (6.2%)-polymer-Ab_EGFR_-treated cells can achieve two-photon luminescence with 71 times less power required for two-photon autofluorescence (1322.8 nJ pixel^−1^ with 500 scans) of similar intensity. This economy can minimize photodamage to cells, rendering amino-N-GQD-polymers suitable for noninvasive 3D bioimaging.

## 1. Introduction

Nanotechnology has been widely studied and applied in almost every field. In the biomedical field, nanotechnology is used for therapy, diagnosis, and detection. Currently, highly stable and biocompatible carbon (C)-based nanomaterials are widely used in bioimaging. Graphene quantum dots (GQDs), which mainly comprise C atoms, exhibit adequate surface grafting that involves π–π conjugations and have expansive surface areas. The formation of surface groups may be attributed to a new phenomenon linked to defect- and intrinsic-state emissions and to a photoluminescence (PL) mechanism [1]. Intrinsic-state emission is induced by the quantum size effect, recombination of localized electron-hole pairs, or zigzag edge sites, whereas defect-state emission results from the defect effects (energy traps). However, GQD-based materials exhibit a low PL quantum yield (QY), which is inadequate for practical application. This drawback substantially limits the application of GQD-based materials in photomedicine.

Atom doping facilitates the modification of the intrinsic properties of GQDs, including their surface features, local chemical features, and electronic characteristics. This process can thus result in GQDs with unique quantum confinement and edge effects, which may be completely distinct from those of unaltered GQDs (i.e., sp^2^-bonded GQDs) [2,3]. The production of nitrogen-doped GQDs (N-GQDs), which can engender heterocyclic aromatic compounds, may alter the chemical composition of GQDs and adjust the GQD band gap to a value that can improve the electrocatalytic, electrochemical, and photochemical properties of GQDs. Thus, achieving tunable luminescence in bioimaging applications becomes feasible. Moreover, the use of primary amine molecules (i.e., amino functionalization) for chemical modification causes substantial electron donation, which considerably enhances the electronic characteristics of N-GQDs [4]. Because N-doped and amino-functionalized GQDs (i.e., amino-N-GQDs) exhibit singlet–triplet splitting, the efficiency of intersystem crossing is sufficient to compensate for the internal conversion in states with identical multiplicity levels. Consequently, the fluorescence emissions may increase [5]. Research has demonstrated that C-based nanomaterials with oligomeric poly(propionylethyleneimine-co-ethyleneimine) and poly(ethylene glycol) diamine surface conjugation exhibit a substantially higher QY and fluorescence than do other C-based nanomaterials because the conjugation impedes the nonradiative recombination of localized hole–electron pairs into sp^2^ clusters, enhances the surface integrity of the π-electron network, and improves electronic properties and optical characteristics [6,7].

Two-photon excitation (TPE) laser microscopy, i.e., multiphoton microscopy, involves the use of a localized nonlinear excitation to induce luminescence. TPE microscopy is a variant of laser-scanning microscopy and has been used in numerous imaging studies [8]. TPE can be combined with near-infrared (NIR) laser excitation to achieve maximum tissue transmission to facilitate bioimaging. NIR radiation exhibits optimal irradiation penetration, reduced photobleaching, low energy absorption, and marginal scattering; such radiation thus facilitates the examination of phantom tissues and deep biological specimens [9]. Several studies have investigated the luminescence caused by continuous-wave lasers under one-photon excitation (OPE). However, the use of illuminated GQD-based nanomaterials as a contrast agent in TPE has not received sufficient attention. Accordingly, to address this gap in the literature, the present study produced N-GQDs through conjugation with an amino group. Characterization results revealed that the produced N-GQDs exhibited desirable photoproperties and that they were effective contrast agents. The study also produced two types of amino-N-GQDs with different compositions of bonded N and amino-functionalized groups (i.e., 4.9% and 6.2%). Assessment results revealed that the amino-N-GQDs with 6.2% of nitrogen and amino-functionalized groups exhibited superior two-photon properties and were more effective contrast agents. Furthermore, this study conducted TPE microscopy in the NIR spectrum to explore the two-photon properties of the two amino-N-GQDs (i.e., amino-N-GQD (6.2%) and amino-N-GQD (4.9%)) and N- and sulfur (S)-containing polymers conjugated with nanomaterials (i.e., amino-N-GQD-based nanomaterial polymers). The findings indicated that compared with the two amino-N-GQDs, the amino-N-GQD-based nanomaterial polymers exhibited superior two-photon luminescence (TPL), and QY (≥0.6) properties, including two-photon absorption (TPA) in the NIR region (800 nm), a large absolute TPE cross section (>59800 Goeppert-Mayer units (GM)), post-TPE stability after 8-min exposure, an average lifetime of approximately 1.11 ns), a favorable ratio of radiative to nonradiative decay rates (>1.50), and TPL. Additionally, the aforementioned polymers exhibited various photoproperties depending on the N dopant and amino group content. In noninvasive 3D two-photon imaging (TPI), the optimal *z*-depth that could be observed using a laser optical system was approximately 105 μm. This could be observed at an ultralow energy of 16.9 nJ pixel^−1^ under 30 scans at an excitation wavelength of 800 nm (total effective illumination, ~0.14 s; scan rate, 4.53 ms per scan; scan area, 200 μm × 200 μm; please see the Materials and Methods section for the calculation). Consequently, the addition of the amino groups and N dopant enabled the GQD-based nanomaterials to become an effective contrast agent for biomedical imaging and tracking.

## 2. Results and Discussion

**Figure 1:**
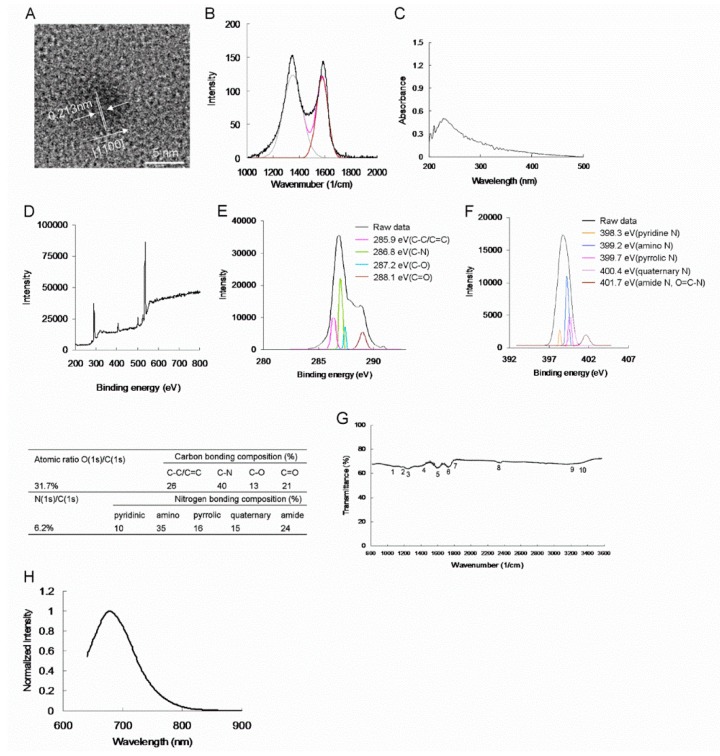
(**A**) HR-TEM image obtained for a single amino-N-GQD (6.2%) that exhibited a 0.213-nm *d*-spacing. (**B**) Raman spectroscopy results indicating the material’s crystallinity. The gray and brown lines indicate that the spectrum was decomposed and fitted into the D-band (~1383 cm^−1^) and G-band (~1605 cm^−1^) peaks (black line: raw data; pink line: decomposed spectrum). (**C**) UV–Vis spectrum of the material. (**D**) Full-range XPS results for the material. (**E**,**F**) Peaks fitted by employing a Gaussian function for the deconvoluted C(1s) and N(1s) XPS spectra. The peaks of a nonoxygenated ring (C–C/C=C) as well as those of the C-N, hydroxyl (C–O), and carbonyl (C=O) bonds were fitted by employing a Gaussian function for the deconvoluted C(1s) XPS spectra. The peaks of pyridinic N (398.2 eV), amino N (NH2), pyrrolic N, quaternary N, and amide N (O=C–N) were fitted by employing a Gaussian function for the deconvoluted N(1s) XPS spectra. The atomic ratios and binding compositions of the amino-N-GQD (6.2%) are summarized in the table. The O(1s)/C(1s) and N(1s)/C(1s) atomic ratios were 31.7% and 6.2%, respectively. (**G**) FTIR spectrum of the material. (**H**) Fluorescence spectrum of amino-N-GQD (6.2%) (Ex/Em: 630 nm/683 nm).

**Figure 2:**
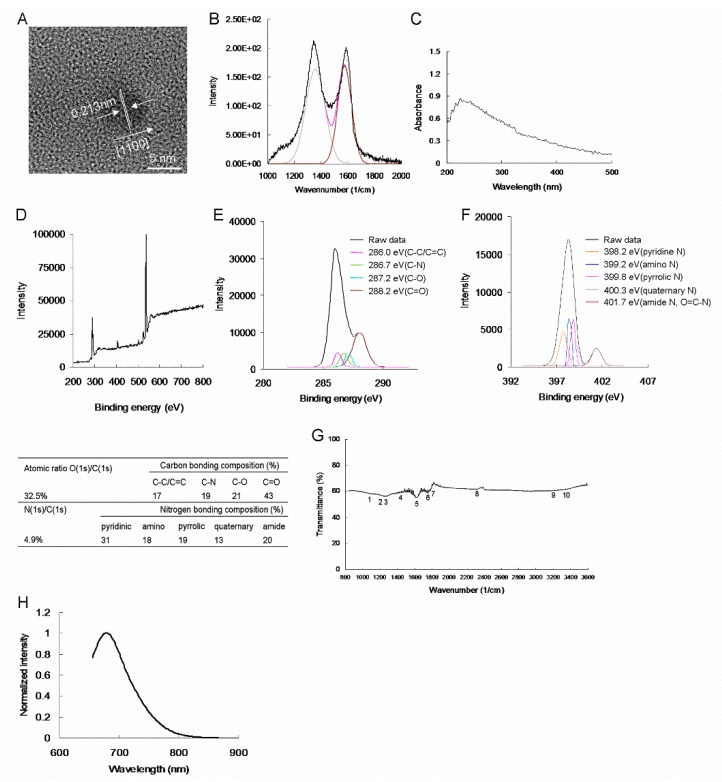
(**A**) HR-TEM image obtained for a single amino-N-GQD (4.9%), which exhibited graphene {1$\overset{-}{1}$00} lattice planes, a mean lateral size of 7.1 ± 0.7 nm, and a *d*-spacing of 0.213 nm. (**B**) Raman spectroscopy results indicating the material’s crystallinity. The gray and brown lines indicate that the spectrum was decomposed and fitted into the D-band (~1385 cm^−1^) and G-band (~1607 cm^−1^) peaks (black line: raw data; pink line: decomposed spectrum). The *I*_D_/*I*_G_ ratio was approximately 0.89. (**C**) UV–Vis spectrum of the material (peak at 223 nm corresponded to the π–π * transition of aromatic C=C bonds and the n–π * transitions of the C–N and C=O shoulder appeared at approximately 326 nm). (**D**) Full-range XPS results for the material. (**E**,**F**) Peaks fitted using a Gaussian function for the deconvoluted C(1s) and N(1s) XPS spectra. The peaks of a nonoxygenated ring (C–C/C=C, 286.0 eV) as well as those of C-N (286.7 eV), hydroxyl (C–O, 287.2 eV), and carbonyl (C=O, 288.2 eV) bonds were fitted using a Gaussian function for the deconvoluted C(1s) XPS spectra. The peaks of pyridinic N (398.2 eV), amino N (NH_2_, 399.2 eV), pyrrolic N (399.8 eV), quaternary N (400.3 eV), and amide N (O=C–N, 401.7 eV) were fitted using a Gaussian function for the deconvoluted N(1s) XPS spectra. The atomic ratios and binding compositions for the amino-N-GQD (4.9%) are summarized in the table. O (1s)/C (1s) and N (1s)/C (1s) atomic ratios were 32.5% and 4.9%, respectively. (**G**) FTIR spectrum of the material. For the amino-N-GQD (4.9%), the results revealed characteristic bands at approximately 1053 cm^−1^ (band 1), corresponding to C—O stretching; approximately 1194 cm^−1^ (band 2), corresponding to C—N stretching; approximately 1228 cm^−1^ (band 3), corresponding to N—C=O stretching; approximately 1421 cm^−1^ (band 4), corresponding to tertiary alcoholic C-OH bending; approximately 1609 cm^−1^ (band 5), corresponding to a C=C ring; approximately 1755 cm^−1^ (band 6), corresponding to N—H bending and amide; approximately 1813 cm^−1^ (band 7), corresponding to C=O stretching; approximately 2349 cm^−1^ (band 8), corresponding to N—H stretching; t approximately 3218 cm^−1^ (band 9), corresponding to C—H stretching; and approximately 3325 cm^−1^ (band 10), corresponding to N—H vibration. (**H**) Fluorescence spectrum of the material (Ex/Em: 630 nm/678 nm).

**Scheme 1:**
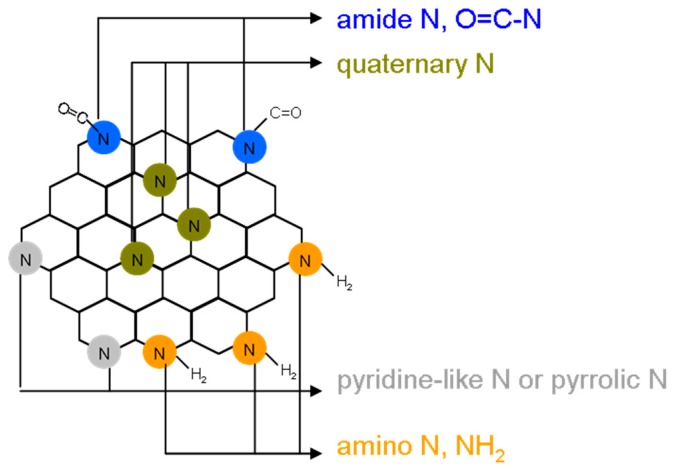
Conceptual schematic of the amino-N-GQD nanomaterial.

GQDs were manufactured using an ultrasonic shearing reaction that incorporated a graphene oxide sheet fabricated through a modified Hummers method [10]. The GQDs were then doped with N, after which they were functionalized with an amino group. High-resolution transmission electron microscopy (HR-TEM) with the accelerating voltage of 120 or 200 keV performed at high magnification revealed that the mean lateral size of the amino-N-GQD (6.2%) was 7.2 ± 0.5 nm (Figure 1A). In addition, satisfactory crystallinity with an appropriate lattice distance was detected; the lattice distance corresponded to the *d*-spacing of graphene {1$\overset{-}{1}$00} lattice fringes. Dynamic light scattering was performed to determine the distribution of material sizes (Supplementary Table S1). In the Raman spectrum, the integrated intensity ratio of the D and G bands (i.e., *I*_D_/*I*_G_ ratio)—located at approximately 1383 and 1605 cm^−1^, respectively—was approximately 0.91, which proved that the produced specimens were of high quality (Figure 1B) [11]. The *I*_D_/*I*_G_ ratios were used as inputs in Raman spectroscopy to estimate the mean size of the sp^2^ domain of the GQD-based specimens [12]. The estimated size approximately matched that obtained from the HR-TEM calculations; nevertheless, the value obtained from the Raman estimation (~6.9 nm) was marginally lower than that obtained from HR-TEM because the oxygenated regions were ignored in the Raman estimation (Equations (1) and (2), Supporting Information) [13,14]. An absorption band was observed at approximately 224 nm and was attributed to the π–π * transition of aromatic C=C bonds. Moreover, n–π * transitions of the C–N and C = O shoulder were observed at approximately 325 nm. This indicates the occurrence of a *π*-electron transition in the amino-N-GQD (6.2%) containing oxygen, demonstrating that the dots were doped with nitrogen, as confirmed through ultraviolet–visible (UV–Vis) spectroscopy (Figure 1C). X-ray photoelectron spectroscopy (XPS) was applied to examine the surface chemistry of nanomaterials, which predominantly contained carbon and nitrogen atoms (Figure 1D). C (1s) spectra were deconvoluted and peaks fitted using a Gaussian function, which revealed the presence of nonoxygenated rings (C–C/C=C, 285.9 eV) as well as C–N (286.8 eV), C–O (287.2 eV), and C=O (288.1 eV) bonds (Figure 1E). N (1s) spectra were also deconvoluted and peaks fitted using the Gaussian function, which revealed pyridinic N (398.3 eV), amino N (NH_2_, 399.2 eV), pyrrolic N (399.7 eV), quaternary N (400.4 eV), and amide N (O = C–N, 401.7 eV) (Figure 1F). The atomic ratios and bonding compositions of the amino-N-GQDs are summarized in the table displayed in Figure 1. The O (1s)/C (1s) and N (1s)/C (1s) atomic ratios were 31.7% and 6.2%, respectively. Fourier transform infrared (FTIR) spectroscopy conducted on the amino-N-GQDs revealed characteristic bands at approximately 1065 cm^−1^ (band 1), corresponding to C-O stretching; approximately 1209 cm^−1^ (band 2), corresponding to C-N stretching; approximately 1241 cm^−1^ (band 3), corresponding to N-C=O stretching; approximately 1438 cm^−1^ (band 4), corresponding to tertiary alcoholic C-OH bending; approximately 1611 cm^−1^ (band 5), corresponding to a C=C ring; approximately 1750 cm^−1^ (band 6), corresponding to N-H bending and amides; approximately 1812 cm^−1^ (band 7), corresponding to C=O stretching; approximately 2336 cm^−1^ (band 8), corresponding to N—H stretching; approximately 3194 cm^−1^ (band 9), corresponding to C—H stretching; and approximately 3338 cm^−1^ (band 10), corresponding to N—H vibration. These FTIR results reveal the presence of exposed amino, epoxy, and hydroxyl groups in the nanomaterials (Figure 1G). Fluorescence characterization was also conducted, and the results are presented in Figure 1H. In addition, the XPS and additional characterization results of the as-prepared amino-N-GQDs (4.9%) are displayed in Figure 2. As displayed in Figure 1, the amino-N-GQD (6.2%) had higher N (1s)/C (1s) atomic ratios and amino and amide N bonding compositions than did the other amino-N-GQD (4.9%), signifying that the substitutional doping of N atoms was caused by the hydrothermal ammonia treatment; moreover, pyridinic, pyrrolic, and quaternary N functionalities were formed. The number of epoxy and carbonyl group moieties converted into amino and amide functionalities was higher for the amino-N-GQD (6.2%) than for the other amino-N-GQD (4.9%). As displayed in Figure 1 and Figure 2, the two types of amino-N-GQDs were successfully realized (Scheme 1).

N dopants can be used to alter the intrinsic properties of GQD-based nanomaterials because the carrier density can be varied such that the electrical and optical characteristics of the modified material differ drastically from those of the original material. Because GQD-based N-doped nanomaterials have unique edge effects and quantum confinement, they exhibit improved electrocatalytic, electrochemical, and photochemical activities. These improvements facilitate the optoelectronic and biomedical operations of GQD-based nanomaterials [15]. Furthermore, the local chemical features and band gaps of graphene structures can be effectively varied through heteroatom doping. Heteroatom doping also alter the electronic and optical properties of GQDs [5]. The QY of N-GQD-based nanomaterials renders them a promising contrast agent for bioimaging. In this study, amino groups were observed on the surface of the produced N-GQDs, which resulted in hole–electron radiative recombination and consequently enhanced intrinsic-state emissions. However, NH_2_ groups were noted at the edges of the N-GQDs and were indicated to have a substantial highest occupied molecular orbital because of the substantial orbital interaction with the primary amine [16]. Thus, the resonance between the delocalized π-orbital and the molecular orbital in the primary amine may result in the narrowing of the orbital band gap, leading to an increase in the fluorescence QY. The calculated relative fluorescence QY of the amino-N-GQD (6.2%) was approximately 0.35; the reference QY, namely QY_ref_, is 0.28 and represents the QY of Cy5.5 in dimethyl sulfoxide (DMSO) [17]. The amino-N-GQD (6.2%) had a higher relative fluorescence QY than did the other amino-N-GQD (4.9%; ~0.31). Moreover, identical QY values were obtained for OPE and TPE [18]. To investigate this in detail, the edge, shape, surface, and shape functionalities of the band gap of amino-N-GQD-based nanomaterials can be manipulated [19]. In addition, surface passivation can enhance the integrity of the surface *π*-electron network, inhibit localized hole–electron pair nonradiative recombination, and increase the QY of amino-N-GQD-based nanomaterials [20]. However, few studies [21,22] have investigated the use of diverse polymers that incorporate S and N and are conjugated with amino-N-GQD-based nanomaterials (amino-N-GQD-based nanomaterial polymers). Accordingly, the present study fabricated polymers that were conjugated with N (polyethylenimine, PEI) and S (polystyrene sulfonate, PSS), namely amino-N-GQD-based nanomaterial polymers. The Amino-N-GQDs were coated with polymers through electrostatic interaction to produce amino-N-GQD-PSS-PEI (or amino-N-GQD-polymers). The polymers were characterized, and the results are illustrated in Figures S1–S3. The amino-N-GQD-polymers with 6.2% and 4.9% N and amino content had relative QYs of 0.69 and 0.60, respectively (QY_ref_ = 0.28, representing the QY of Cy5.5 in DMSO [17]).

**Figure 3:**
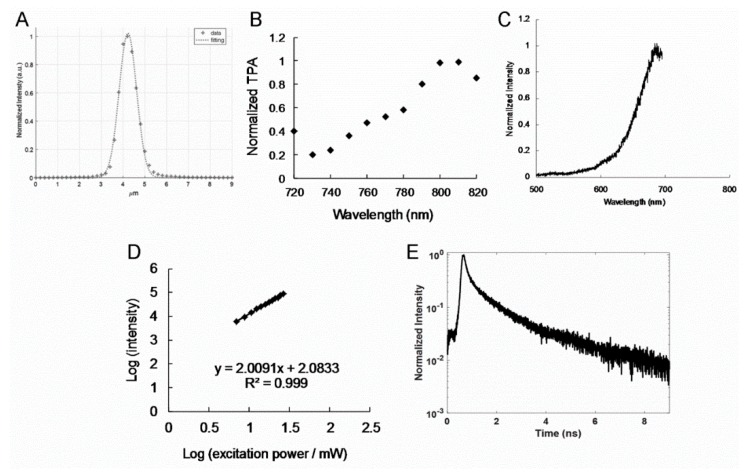
(**A**) According to the *z*-axis scan of a gold thin film for measuring the signal of second harmonic generation at different positions, the *z*-axis resolution of the laser system (full width at half maximum) was approximately 0.94 μm (fitting performed using the Gaussian function). (**B**) Relative TPA spectrum of the amino-N-GQD (6.2%)-polymers. Signals were observed through TPE at various wavelengths (720–820 nm) at 70.4 nJ pixel^−1^. (**C**) Relative TPL spectrum of the amino-N-GQD (6.2%)-polymers under TPE (Ex/Em: 800/689 nm; 70.4 nJ pixel^−1^; cutoff of 700 nm, as determined through cascading filters). (**D**) Dependence of TPL intensity on the nanomaterial excitation power (logarithm), with the corresponding slope being approximately 2.01. The nanomaterial was subjected to TPE at 704.0–2816.0 nJ pixel^−1^ (R^2^ ≥ 0.999, Ex: 800 nm). (**E**) Time-resolved TPL material decay profiles observed at room temperature (Ex: 800 nm, 70.4 nJ pixel^−1^). The delivered dose of the material was 4 μg mL^−1^ in ddH_2_O.

**Figure 4:**
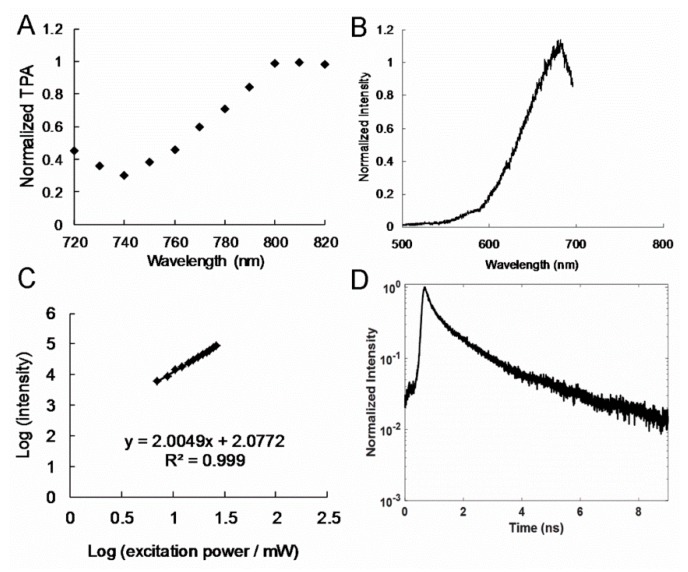
(**A**) Relative TPA spectrum of the amino-N-GQD (4.9%)-polymers. Signals were observed through TPE at various wavelengths (720–2820 nm) at 70.4 nJ pixel^−1^. (**B**) Relative TPL spectrum of the amino-N-GQD (4.9%)-polymers obtained under TPE (Ex/Em: 800 nm/682 nm; 70.4 nJ pixel^−1^; cutoff of 700 nm as determined through cascading filters). (**C**) Dependence of TPL intensity on nanomaterial excitation power (logarithm), with the corresponding slope being approximately 2.00. The nanomaterial was subjected to TPE at 704.0–2816.0 nJ pixel^−1^ (R^2^ ≥ 0.999; Ex: 800 nm). (**D**) Time-resolved TPL material decay profiles observed at room temperature (Ex: 800 nm, 70.4 nJ pixel^−1^). The delivered dose of the material was 4 μg mL^−1^ in ddH_2_O.

TPE was conducted at a laser power of 70.4 nJ pixel^−1^ (see the Materials and Methods Section 3.8 for details regarding the laser power calculations). The focal spot resolution of the laser system in the z-axis was 0.94 μm (Figure 3A) and that in the *x−y*-axes was 0.38 μm. Thus, the laser power was maintained at a low level, and the excitation wavelength was lengthened to the NIR region. Two-photon characteristics were determined through nonlinear multiphoton laser excitation in the NIR spectrum. The results revealed that the GQD-based nanomaterial polymers had a satisfactory TPA at 800 nm in the NIR spectrum (Figure 3B and Figure 4A), and this could be attributed to the employed interband transition [23], which enabled the detection of a deep specimen or tissue. TPL spectra were measured for the polymers. The peaks observed for the amino-N-GQD (6.2%)-polymers were at approximately 689 nm (Figure 3C), and those observed for the amino-N-GQD (4.9%)-polymers were at nearly 682 nm (Figure 4B), when TPE was performed at 800 nm, which is comparable to the OPE wavelength in Figure 1H and Figure 2H. In a two-photon process, the PL intensity has a quadratic dependence on the excitation power under TPE (Ex: 800 nm) [24], with an exponent of approximately 2.01 and 200 (Figure 3D and Figure 4C). The large absolute cross section of TPE renders chromophores suitable for exploration and effective for in vitro or in vivo examination of molecular activities through two-photon approaches. The chromophores are rendered suitable for the aforementioned activities because they correspond to a high ratio of the energy absorbed by a specimen to its input energy flux, which reduces the likelihood of photodamage. In this study, the absolute cross sections of TPE for the amino-N-GQD (6.2%)-polymers and amino-N-GQD (4.9%)-polymers were approximately 61176 and 59837 GM (Table 1), respectively (1 GM = 10^−50^ cm^4^ s photon^−1^; rhodamine B was selected as the reference [25] for producing the cross section). The lifetime of the nanomaterials was measured (Figure 3E) by fitting the results of time-correlated single-photon counting with a triple-exponential function. The results indicated that the amino-N-GQD (6.2%)-polymers had a mean lifetime of approximately 0.97 ± 0.02 ns (the observed lifetimes were 0.15 ± 0.01, 0.87 ± 0.02, and 3.70 ± 0.16 ns). By contrast, the mean lifetime of the amino-N-GQD (4.9%)-polymers was approximately 1.11 ± 0.04 ns (Figure 4D and Table 2). The two-photon characteristics of the GQD-based nanomaterial polymers could be attributed to the reduced lifetime, which was probably caused by the increased quantum confinement of the emissive energy on the surfaces of the GQD-based nanomaterials (each particle possessed a large surface-to-volume ratio). Furthermore, the ratio of radiative to nonradiative decay rates was approximately 2.22 (7.11 × 10^8^/3.20 × 10^8^ s^−1^) for the amino-N-GQD (6.2%)-polymers and approximately 1.50 (5.41 × 10^8^ s^−1^/3.60 × 10^8^ s^−1^) for the amino-N-GQD (4.9%)-polymers. These findings indicate that the nanomaterials traversed the radiative pathway rather than the nonradiative pathway following TPE when the lifetime decreased and QY increased. The effective TPL radiative emission can be attributed to the amino functionalization and doping of GQDs with bonded N atoms. This is because sp^2^ hybridization can be restored and delocalized electrons in the π * states can be donated, thus increasing the absolute TPE cross section. Moreover, the increased charge transfer efficiency can be attributed to the influence of strong electron donation and large π-conjugated systems in the polymers incorporating N and S atoms that were coated onto the amino-N-GQDs. The interaction between the amino-N-GQD-polymers produced oscillating dipoles [26]. Therefore, the two-photon characteristics of these N-conjugated polymers changed abruptly [16]. These phenomena are invaluable for two-photon examinations and TPI in particular, as demonstrated by this study.

**Figure 5:**
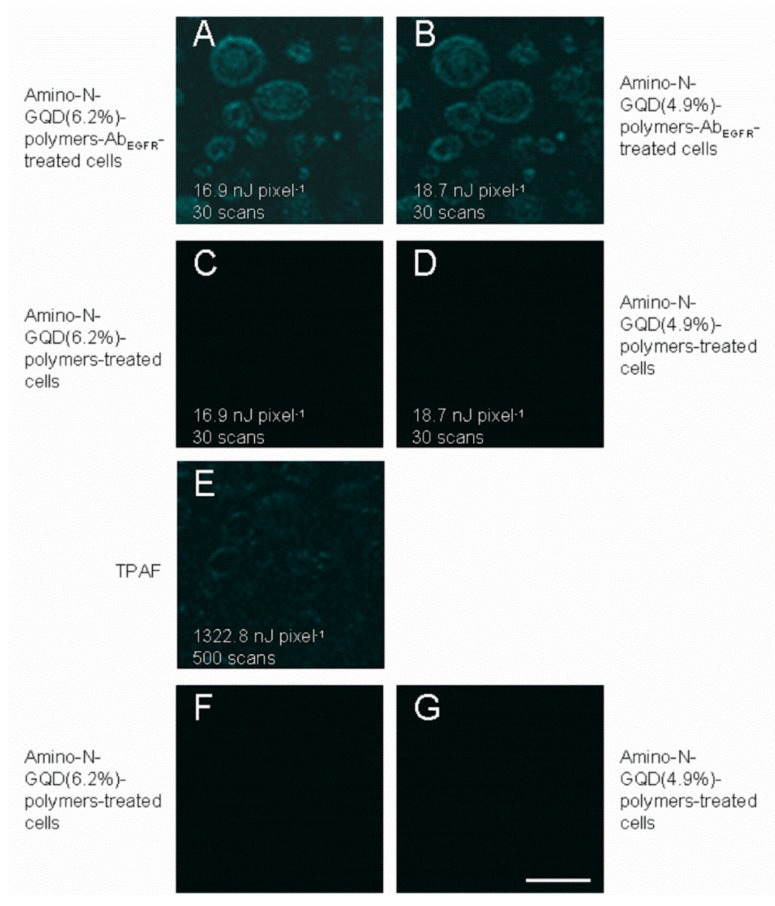
TPL images (cyan) derived at an imaging depth of 105 μm. (**A**) Amino-N-GQD (6.2%)-and (**B**) amino-N-GQD (4.9%)-Ab_EGFR_-treated cells observed at TPE power levels of 16.9 and 18.7 nJ pixel^−1^ over 30 scans, respectively. (**C**,**D**) Amino-N-GQD-polymer-treated cells not coated with an antibody underwent the same treatment. (**E**) TPAF images of the unlabeled cells (cells with no treatment materials) were captured at a TPE power of 1322.8 nJ pixel^−1^ over 500 scans. (**F**,**G**) Images of the amino-N-GQD-polymer-Ab_EGFR_-treated cells at the same depth with an OPE power of 9 mW. The delivered dose of the material was 4 μg mL^−1^ in cell culture medium; the excitation wavelength was 800 nm; and the scale bar is 20 μm.

**Figure 6:**
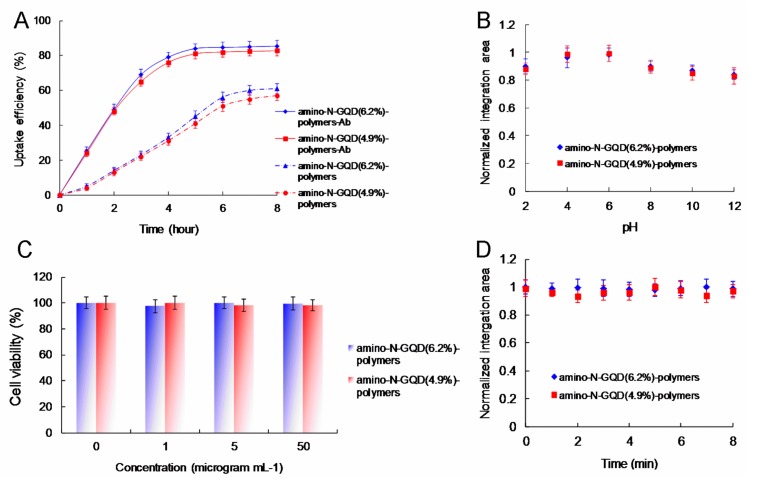
(**A**) Uptake assay results derived for cells treated with nanomaterials for 0–8 h at 37 °C. (**B**) pH-dependent TPL emission of nanomaterials subjected to a TPE power of 2816 nJ pixel^−1^ (Ex: 800 nm). The TPL intensity observed for the integrated area was a function of pH (2–12). The intensity ranged from 500 to 695 nm. (**C**) Viability (24 h) of nanomaterial-treated cells obtained through a 3-(4,5-dimethylthiazol-2-yl)-2,5-diphenyltetrazolium bromide (MTT) assay. (**D**) Photostability of nanomaterials subjected to a TPE power of 2816 nJ pixel^−1^ for 8 min (approximately 105960 scans; Ex: 800 nm). The emission intensities of the integrated area after photoexcitation were divided by those of the newly prepared nanomaterial without photoexcitation to obtain the normalized integrated area. The delivered dose of the material was 4 μg mL^−1^ dissolved in cell culture medium, PB buffer or ddH_2_O. Data are represented as mean ± SD (*n* = 6).

**Figure 7:**
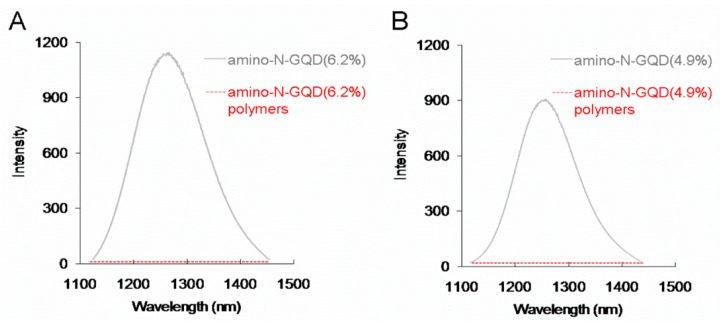
Phosphorescence spectra at 1270 nm for the (**A**) amino-N-GQD (6.2%)- and (**B**) amino-N-GQD (4.9%)-polymers. The delivered dose of the material was 4 μg mL^−1^ in ddH_2_O.

**Figure 8:**
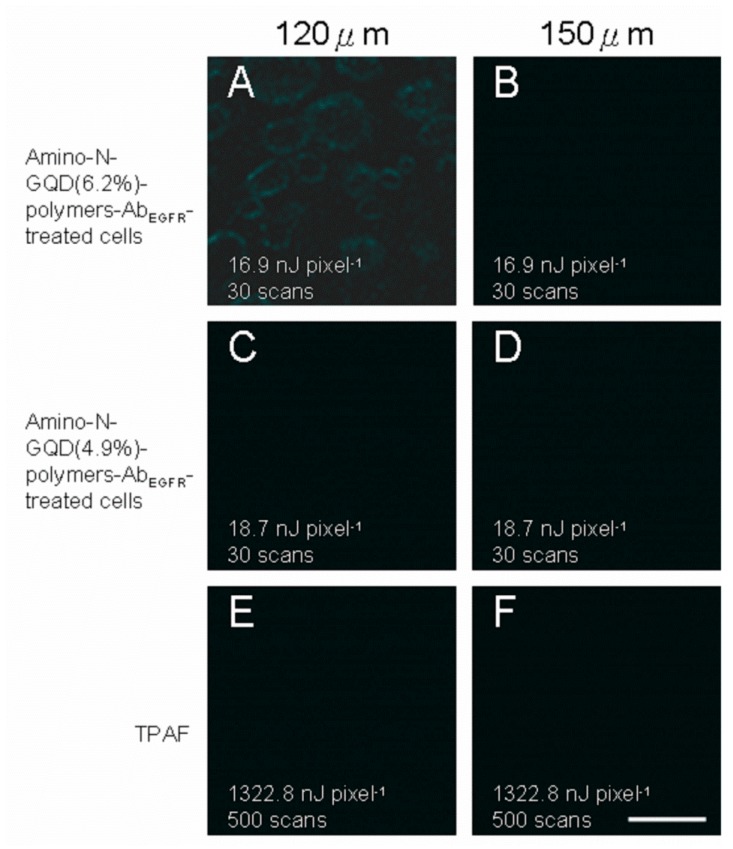
(**A**–**D**) TPI results (cyan in color) of the nanomaterial-polymer-Ab_EGFR_-treated cells and (**E**,**F**) TPAF imaging results of the unlabeled cells at different depths ranging from 125 to 150 μm (with a constant increment) and TPE powers of 16.9, 18.7 nJ pixel^−1^ (30 scans), and 1322.8 nJ pixel^−1^ (500 scans) (Ex: 800 nm). The delivered dose of the material was 4 μg mL^−1^ in cell culture medium, and the scale bar is 20 μm.

The superior two-photon properties under TPE conditions indicate that both types of amino-N-GQD-polymers used in this study constitute excellent contrast agents with considerable potential for use in noninvasively identifying deep and 3D biological specimens using a TPE wavelength extendable to the NIR spectrum. As presented in Figure 3B and Figure 4A, the brightest TPL signal produced by the two nanomaterial polymers and the brightest two-photon autofluorescence (TPAF) signal produced by cancer cells were observed at an excitation wavelength of 800 nm. Cancer cells comprise biological molecules producing TPAF signals [27]. A home-fabricated optically inverted microscopy system is unsuitable for use in vivo assay processes. Consequently, to determine the effectiveness of TPL signals for conducting TPI on deep tissue, cells embedded in a collagen matrix, imitating 3D epithelial tissue, were used in this study (Supporting Information) [28]. TPL images of A431 skin cancer cells treated using nanomaterials conjugated with an EGFR antibody (nanomaterial-Ab_EGFR_-treated cells) were captured at an imaging depth of 105 μm under TPE (Ex: 800 nm), as depicted in Figure 5. The cells overexpressed cell-surface EGFR. Burst uptake rates of 69.2% and 67.5% were observed for the amino-N-GQD (6.2%)-polymers and amino-N-GQD (4.9%)-polymers treated with Ab_EGFR_, respectively, within 3 h. The burst uptake rates of the aforementioned polymers increased to approximately 85.4% and 82.7% in their final profiles, respectively (Figure 6A). Moreover, the amino-N-GQD-polymers had similar trends to the amino-N-GQD-polymer-Ab_EGFR_ but exhibited lower efficiency, indicating that high quantities of nanomaterial-Ab_EGFR_ were absorbed onto the cellular surfaces. This finding was confirmed through an uptake assay. The nanomaterial-Ab_EGFR_-treated cancer cells exhibited oxidative stress independent of reactive oxygen species (ROS) under TPE, as determined using singlet oxygen sensor green (SOSG), trans-1-(2′-methoxyvinyl)pyrene (*t*-MVP), 2,3-bis (2-methoxy-4-nitro-5-sulfophenyl)-2H-tetrazolium-5- carboxanilide (XTT), and glutathione (γ-L-glutamyl-L-cysteinyl-glycine, GSH) probes (Table 3 as well as Supplementary Tables S2 and S3) [29,30,31,32,33,34,35]. The aforementioned result was consistent with the signal of ^1^O_2_ phosphorescence at 1270 nm emitted from the amino-N-GQD-polymers (Figure 7). Furthermore, the measured singlet oxygen quantum yields (ψ_△_) of the amino-N-GQD (6.2%), amino-N-GQD (6.2%)-polymers, amino-N-GQD (4.9%), and amino-N-GQD (4.9%)-polymers were approximately 0.56, 0.50, 0.02, and 0.01, respectively (ψ_△_= 0.64 is the QY of *meso*-tetra(4-sulfonatophenyl)porphine dihydrochloride in deuterium oxide [36]), indicating that polymer coating can inhibit the release of generated ROS; this thus renders nanomaterials favorable candidates for stable imaging probes. Both amino-N-GQD-polymers produced in this study were determined to be suitable for achieving high TPL in acidic environments (such as those of cancerous specimens or tissues) due to their favorable photostability (Figure 6B). To achieve favorable stability in the physiological environment of amino-N-GQD-polymer-Ab_EGFR_-treated cells (Figure 6C and Table S4), TPI should be conducted at TPE power levels of 16.9 and 18.7 nJ pixel^−1^ after 30 scans (total effective illumination, ~0.14 s; scan time, 4.53 ms^1^; and scan area, 200 μm × 200 μm; please see the Materials and Methods section for the calculation) at an imaging depth of 105 μm to maintain the TPL intensity. This represents a reduction in detected power emission compared with that observed for cells treated with GQD polymers with lower nitrogen and amino-functionalized group compositions and conjugated with Ab_EGFR_. The two-photon images of cells treated with the two types of nanomaterial polymers conjugated with Ab_EGFR_ (Figure 5A,B) were not as clear as those of cells treated with the two amino-N-GQD-polymers (without conjugation of antibodies) at a depth of 105 μm under same exposure (Figure 5C,D). Moreover, the TPAF images (500 scans with a total effective illumination of approximately 2.27 s, Figure 5E) of the intrinsic fluorophores in the cancer cells corresponded to a TPE power of 1322.8 nJ pixel^−1^ in unlabeled cells to acquire an identical signal level. The emission intensity exhibited a quadratic dependence on the incident power, which implies that for the same intensity and excitation power, the excitation ratio between the TPI modalities of the amino-N-GQD-polymer-Ab_EGFR_-treated cells was at least 71 times less than that required for the TPAF imaging of unlabeled cells. This suggests that the TPL intensity was approximately 5040 times the TPAF intensity for the unlabeled cells. Moreover, the amino-N-GQD-polymers exhibited two-photon stability, as indicated by the TPL intensity determined after TPE, which resulted in a reduced photobleaching effect (Figure 6D). The results indicate that TPI was not negatively affected by spherical aberrations arising from mismatches between the immersion oil and TPE sample in terms of refractive index, even at a depth of 105 μm. By contrast, the emissions of the amino-N-GQD-polymer-Ab_EGFR_-treated cells were not effectively detected at large depths (Figure 5F,G) under an OPE power of 9 mW (FV1000, HeNe-R 633 nm, Gas, 10 mW; 40× oil-immersion objective (*NA*: 1.3), Olympus, Shinjuku, Tokyo, Japan). The results also indicate that imaging could be performed up to the lens’ working distance for both phantom tissues under TPL and TPAF. For each 30-μm increase in TPI depth up to 150 μm under TPE (Ex: 800 nm), TPL images of the nanomaterial-Ab_EGFR_-treated cells and TPAF images of the unlabeled cells were recorded (Figure 8). Figure 8A reveals slight contour of cells for having the best two-photon properties among Figure 8. However, the images acquired from depths exceeding 120 μm were severely and negatively affected by spherical aberrations because of the detection efficacy, employed objective, maximal *z*-depth recorded using the optical laser setup, and mismatch between the aqueous sample and immersion oil in terms of refractive index.

## 3. Materials and Methods

### 3.1. Preparation of Amino-N-GQDs 

Graphene oxide was prepared from a natural graphite powder (Bay carbon Inc., Bay City, MI, USA) using a modified Hummers’ method [10,37,38,39,40]. Graphite (8.5 M) and NaNO_3_ (0.6 M) (Merck & Co., Kenilworth, NJ, USA) were mixed with H_2_SO_4_ (FUJIFILM Wako Chemicals USA Inc., Richmond, VA, USA). KMnO_4_ (2.0 M) (Fisher Scientific, Hampton, NH, USA) was slowly added with continual stirring at 35 °C overnight. Subsequently, deionized water (ddH_2_O) was gradually added and continually stirred. Adding H_2_O_2_ (Sigma Aldrich Co., St. Louis, MO, USA) was the method used to terminate the reaction. Washing and centrifugation with ddH_2_O several times were carried out, and the graphene oxide was collected. The as-prepared graphene oxide was placed in a tube furnace and heated to 400–600 °C [400 °C and 600 °C for amino-N-GQD (4.9%) and amino-N-GQD (6.2%) of the N(1s)/C(1s) ratio determined by XPS, respectively] in the presence of ammonia for 4–6 h. N-GQDs were subsequently obtained following the same procedure. The as-prepared N-GQDs were mixed with ammonia (Sigma Aldrich Co., St. Louis, MO, USA), stored in a Teflon-lined stainless-steel autoclave, and reacted at 180 °C for 5 h. The resulting mixture was washed with ddH_2_O, centrifuged several times, and subsequently dried in an oven at 50 °C overnight. Eventually, amino-N-GQDs were obtained.

### 3.2. Synthesis and Characterization of Amino-N-GQD-Polymers

Both negatively charged PSS and positively charged PEI (both 40 μg mL^−1^; Sigma Aldrich Co., St. Louis, MO, USA) were coated on the surface of as-prepared positively charged amino-N-GQDs (40 μg mL^−1^) through electrostatic interaction to form amino-N-GQD-PSS-PEI (or amino-N-GQD-polymers). The solutions were centrifuged (82,000 rpm, Optima TLX Ultracentrifuge, Beckman Coulter Inc., Danville, CA, USA) for 20 min to remove excess polymers. The pellets (amino-N-GQD-polymers) were re-suspended in ddH_2_O, and the centrifugation process was repeated several times.

### 3.3. Characterization

Materials were subject to TEM (JEOL 2100F and JEOL 3010, Akishima, Tokyo, Japan) observation at the accelerating voltage of 120 or 200 keV. FTIR spectroscopy, UV-Vis, and zeta potential spectra of samples were recorded by the spectrometers: RX1, PerkinElmer, Waltham, MA, USA; U-4100 Hitachi, Chiyoda-ku, Tokyo; and Malvern Nano-ZS90, Worcestershire, West Midlands, UK, respectively. Raman spectroscopy (DXR, Thermo Scientific, Waltham, MA, USA) was used to examine the crystallinity of samples with 532 nm laser. XPS (PHI 5000, VersaProbe, Chanhassen, MN, USA) was employed to examine the surface chemistry of the materials, the O (1s)/C (1s) and N (1s)/C (1s) atomic ratios of materials. The fluorescence signal was recorded by the spectrophotometer (F-7000, Hitachi, Chiyoda-ku, Tokyo, Japan).

### 3.4. Data of XPS

Amino-N-GQD (6.2%)-polymers: C–C/C=C (285.9 eV), C–N (286.8 eV), C–O (287.2 eV) and C=O (288.1 eV). Amino-N-GQD (4.9%)-polymers: nonoxygenated ring (C–C/C=C, 286.0 eV), C-N bonds (286.7 eV), hydroxyl (C–O, 287.2 eV), and carbonyl (C=O, 288.2 eV). The C–O bonds were identified as corresponding to functional groups of epoxy and tertiary alcohol on the basal plane as well as phenol and ether groups located at the graphene sheet’s periphery. The C=O bond indicated the presence of carbonyl group at the graphene periphery. The contribution of the C–N bonds was increased at the expense of those of the C–O and C=O bonds through ammonia treatment. This indicated that the epoxy and carbonyl groups were converted in the treatment. The deconvoluted N(1s) spectrum of the amino-N-GQD (6.2%)-polymers provides further details of the C–N bonding, and it is indicated that the ammonia-treated samples contained pyridinic (398.3 eV), amino (399.2 eV), pyrrolic (399.7 eV), quaternary (400.4 eV), and amide (O=C–N, 401.7 eV) nitrogen-containing functional groups [Amino-N-GQD (4.9%)-polymers: pyridinic N (398.2 eV), amino N (NH_2_, 399.2 eV), pyrrolic N (399.8 eV), quaternary N (400.3 eV), and amide N (O=C–N, 401.7 eV)]. Derived from the deconvoluted N(1s) spectra, the nitrogen functionality compositions indicated that the hydrothermal ammonia treatment resulted in considerable nitrogen atom doping and led to the formation of pyridinic, pyrrolic, and quaternary nitrogen functionalities. Some proportions of epoxy and carbonyl groups were also discovered to have been converted; they respectively formed amino and amide functionalities.

Nitrogen functionalities are known to strongly affect the resonance patterns of electron orbitals in all materials based on graphene oxide. The annealing ammonia treatment may have caused aromatic ring damage and the formation of defective pyrrolic and carbonyl groups at the periphery as the graphene oxide treated with ammonia was ultrasonically exfoliated and cut in nitric acids. The subsequent hydrothermal ammonia treatment of the produced N-GQDs caused conversion of the carbonyl groups to amide groups on amino-N-GQDs. The corresponding conceptual schematic of the amino-N-GQD is shown in Scheme 1.

### 3.5. Coating Antibodies

The absorbance of a certain quantity of Ab_EGFR_ (Abcam, Cambridge, MA, USA) was recorded via UV-Vis spectroscopy (Abs: approximately 270 nm and 394 nm) (Supporting Information, Figure S4). By electrostatic interaction, the materials were mixed with the same quantity antibody for 30 min during incubation at 4 °C in the dark and centrifuged (83,000 rpm) to remove excess antibody; the nanomaterial-Ab_EGFR_ was then prepared. Conversely, the supernatant was retained, and its absorbance was measured. The difference between the absorbance of the collected supernatant and the original antibody was estimated. Consequently, the quantity of the antibody absorbed on the materials was calculated using Beer–Lambert law (*A* = *εbC*, where *A* = absorbance, *ε*= molar extinction coefficient, *b* = path length (1 cm), and *C* = concentration). In the culture medium of human squamous carcinoma cell line (A431 skin cancer cells), approximately 9.3 µg of Ab_EGFR_ coated on 100.0 µg of amino-N-GQD (6.2%)-polymers. This implies that the absorption efficiency culture medium of A431 cancer cells was approximately 9.3% (zeta potential of amino-N-GQD (6.2%)-polymers-Ab_EGF_: 12.8 eV). For amino-N-GQD (4.9%)-polymers, the efficiency of absorption was approximately 9.0% (zeta potential of amino-N-GQD-Ab_EGFR_: 12.5 eV). The above results prove the successful coating of Ab on the surface of materials.

### 3.6. Cell Culture of A431 Skin Cancer Cells and MTT Assay

A431 cells were cultured in EMEM (EBSS) + 2mM Glutamine + 1% Non Essential Amino Acids + 10% Fetal Bovine Serum at 37 °C under 5% CO_2_ in air. The cells were collected by trypsinization and placed onto a 10 cm tissue culture Petri dish, then allowed to grow for 2–4 days. For MTT assay, cells (5 × 10^3^ per well in a 96-well culture plate) were incubated overnight and then treated with material-Ab_EGFR_ for 24 h incubation in an incubator (37 °C). Cell viability was evaluated using MTT assay. A431 cells were seeded into the 96-well culture dish plates contain 150 μL of the culture medium for 24 h. Before the assay, the materials were mixed into the culture medium at concentrations ranging from 0 to 50 μg mL^−1^. After 24 h, 150 μL of MTT solution (1 mg 150 mg mL^−1^) was added for 4 h reaction with the cells at 37 °C. After removal of the medium and MTT solution, 150 μL of DMSO was added to each well, and the assay plate was read at optical density 595 nm with an ELISA reader (Thermo Electron, Waltham, MA, USA). The absorbance of the untreated cells in the control group was considered 100%.

### 3.7. QY Measurement

The relative fluorescence QY of contrast agent is the usually the ratio of the emitted photons to the absorbed photons and is given as follows:

QY = QY_ref_ (*η*^2^/*η*_ref_^2^)(*I*/*A*)(*A*_ref_/*I*_ref_) (1)

where QY_ref_ = 0.28 is the QY of Cy5.5 dissolved in DMSO as a reference, *η* is the refractive index of ddH_2_O = 1.33 (*η*_ref_ of DMSO = 1.48), *I* is the integrated fluorescence intensity and *A* is the absorbance at the excitation wavelength. OPE or TPE yields the same QY [17,21].

### 3.8. Femtosecond Laser Optical System for the Measurements of TPA and TPL 

The Ti:sapphire femtosecond laser (Tsunami, Spectra-Physics, Santa Clara, CA, USA) optical system with a pulse width of less than 100 fs and a repetition rate of 80 MHz, an inverted optical microscope (Axiovert 200, Zeiss, Oberkochen, Germany), a *x-y* galvanometer scanner (6215H, Cambridge, MA, USA), a triple-axis sample-positioning stage (ProScan^TM^II, Prior Scientific Instruments Ltd., Cambridge, UK), a *z*-axis piezoelectric nano-positioning stage (Nano-F100, Mad City Labs, Madison, WI, USA), an acousto-optic modulator (AOM) (Neos 23080-x-1.06-LTD, BMI Surplus Inc., Hanover, MA, USA), photomultiplier tubes (PMTs) (H5783P, Hamamatsu, Shizuoka Prefecture, Japan), and a data acquisition (DAQ) card with a field-programmable gate array (FPGA) module (PCI-7831R, National Instruments, Austin, TX, USA). The optical system was used according to the previous studies [29,41,42,43].

With a galvanometer scanner speed of 2 m ms^−1^, the excitation spectrum was measured as 720–820 nm with an excitation power of 2.0 mW [this is the power before objective; the power after objective (or on sample) is 0.704 mW or 70.4 nJ pixel^−1^]. Therefore, the relative TPA spectra as function of excitation wavelength for the amino-N-GQD (6.2%)-polymers and amino-N-GQD (4.9%)-polymers were measured.

Measurement of TPL spectrum: The material was exposed to TPE from the femtosecond laser at an excitation wavelength of 800 nm, a power of 70.4 nJ pixel^−1^, a scanning area of 200 μm × 200 μm, a frequency of 10 kHz, an exposure time of 1.638 s/(scan, pixel) = 100 μs, 128 × 128 pixels scan^−1^, and a pixel area of 1562.5 nm × 1562.5 nm. The focal spot area was calculated as π*d*^2^/4, where *d* = 0.61 λ/numerical aperture (*NA*) is the full width at half maximum of the beam waist. For instance, at the *x*–*y* axis focal spot with 800 nm excitation and a 40× oil-immersion objective with an *NA* of 1.3, *d* = 0.61 × 800 nm/1.3 = 375.38 nm = approximately 0.38 μm, and the *z*-axis resolution was measured to be approximately 0.94 μm. For 800 nm excitation, the exposure time per scan for an individual nanomaterial is expressed as (focal spot area/pixel area) × 100 = 4.53 ms, and the total exposure time *t* = 4.53 ms × number of scans. A 40× oil-immersion objective (*NA* 1.3) was used to collect the signals, and the detection range of the spectrum photometer was 300–695 nm.

Additionally, the calculations of laser power (mW or nJ pixel^−1^) used on the sample were as follows. For the 40× oil-immersion objective (*NA* 1.3), the transmission rate at 800 nm in wavelength is approximately 88% in this optical system, and the laser power went from the output to the objective with only 40% of the original output power due to the loss of power. As a result, the calculated energy after the objective (on sample) is *P*_output_ (mW) * 40% * 88% = 0.352 * *P*_output_ (mW). For instance, *P*_output_ = 3.0 mW, the calculated energy after the objective (on the sample) is 3.0 mW*40%*88% = 1.056 mW. With 10 kHz of scan rate (each pulse stays 0.1 ms pixel^−1^), the calculated energy on the sample (J pixel^−1^) was around *P*_output_ (mW)*40% * 88% * 0.1 ms = 0.0352 * *P*_output_ (J pixel^−1^). For instance, *P*_output_ = 3.0 mW, the energy (J pixel^−1^) on sample = 3.0 mW*40% * 88% * 0.1 ms = 0.1056 μJ pixel^−1^ = 105.6 nJ pixel^−1^. The power after the objective (on the sample) was used and marked throughput this manuscript.

### 3.9. Measurement of TPE Absolute Cross Section 

The absolute cross section of TPE was measured on the basis of luminescence signals by using a femtosecond laser optical system, as described by the previous studies [9,16,17,18,20,24,28,29,43,44,45,46,47,48,49,50,51]. The TPL of fluorescein and rhodamine B (Sigma-Aldrich, St. Louis, MO, USA) had to be verified. The results are shown in Figure S5 (Supporting Information) and were obtained by measuring the dependence of the emission intensity with an excitation power range of 704 nJ pixel^−1^ to 2816 nJ pixel^−1^. Quadratic dependence with the exponents of 1.98 for fluorescein and 2.01 for rhodamine B was measured for increasing the excitation power to determine the luminescence from TPE. According to the previous studies, the action cross sections of TPE for fluorescein and rhodamine B are 36.4 and 153.0 Göeppert–Mayer units (GM; 1 GM = 10^−50^ cm^4^s photon^−1^), respectively, for 800 nm excitation. We also referred to the free website http://www.drbio.cornell.edu/cross_sections.html, kindly provided by Prof. Chris Xu (Cornell University, NY, USA). The TPE action cross sections for fluorescein and rhodamine B were calculated to be 37.2 and 155.6 GM, respectively (Supplementary Table S5), which indicated an error of less than 5% compared with those from Prof. Xu’s laboratory. In this study, rhodamine B was chosen as the standard reference for determining the cross section, and the calculated absolute cross sections of TPE for the amino-N-GQD (6.2%)-polymers and amino-N-GQD (4.9%)-polymers were approximately 61176 GM and 59837 GM, respectively. The measured parameters for calculating the TPE absolute cross sections of samples are shown in Table 1. No batch-to-batch variation was observed for the materials in two-photon properties, two-photon photodynamic ability, and two-photon contrast agents.

### 3.10. Femtosecond Laser Optical System (for Fluorescence Lifetime Imaging Microscopy, FLIM) 

The home-made femtosecond Ti:sapphire laser optical system (repetition rate of 80 MHz) (Tsunami, Spectra-Physics, Santa Clara, CA, USA) and FLIM were used according to the previous study [24]. The lifetime data and parameter were generated by the triple-exponential equation fitting (3 exp fitting model: (a0 * exp(a1x) + a2 * exp(a3x) + a4 * exp(a5x) + a6)) while monitoring the emission under TPE (Ex: 800 nm).

### 3.11. Calculation of Radiative and Non-Radiative Decay Rates 

Fluorescence QY and lifetime are both major parameters when investigating the emission characteristics of fluorescent dyes in diverse environments. The quantum yield Q can be expressed as follows:

(2) $$Q=\frac{\Gamma }{\Gamma +k}$$

where Γ is the radiative decay rate and *k* is the nonradiative decay rate [17,48].

Fluorescence lifetime is usually defined as the average time required for an electron in the excited state to decay to the ground state. The TPL lifetime *τ* can also be relative to the decay rates and described as follows:

(3) $$\tau =\frac{1}{\Gamma +k}$$

Following Equations (2) and (3), the radiative and nonradiative decay rates can be calculated [17,48]. The relevant description is provided in the Supporting Information.

### 3.12. Reactive Oxygen Species (ROS) Detection 

Singlet oxygen (^1^O_2_): (a) Material-Ab (4 µg mL^−1^) was treated with A431 cells, after which it was subjected to 3 h of incubation at 37 °C in darkness. Subsequently, the mixture was exposed to TPE photoexcitation (2816 nJ pixel^−1^, 500 scans; Ex: 800 nm) and finally mixed with Singlet Oxygen Sensor Green (SOSG) reagent (1 μM; Thermo Fisher Scientific, Waltham, MA, USA) (Ex/Em: 488/525 nm). A fluorescence spectrometer was employed for measurements. For ROS neutralization, the mixture was mixed with 30 ppm of antioxidant α-tocopherol/methyl linoleate (Sigma-Aldrich, St. Louis, MO, USA) in darkness and exposed to TPE photoexcitation with the same treatment. (b) Material-Ab (4 µg mL^−1^) was treated with A431 cells, after which it was subjected to 3 h of incubation at 37 °C in darkness. Subsequently, the mixture was exposed to TPE photoexcitation (2816 nJ pixel^−1^, 500 scans; Ex: 800 nm) and finally mixed with 10 μM of trans-1-(2’-methoxyvinyl)pyrene (*t*-MVP) (Thermo Fisher Scientific, Waltham, MA, USA)/0.10 M SDS (Sigma-Aldrich, St. Louis, MO, USA) (Ex/Em: 352/465 nm). For ROS neutralization, the mixture was mixed with 30 ppm of antioxidant α-tocopherol/methyl linoleate (Sigma-Aldrich, St. Louis, MO, USA) in darkness. Reaction of *t*-MVP with ^1^O_2_ yields a dioxetane intermediate that fluoresces while it decomposes into 1-pyrenecarboxaldehyde. Furthermore, this highly selective fluorescent probe does not react with other activated oxygen species such as hydroxyl radicals, superoxide, or hydrogen peroxide. A fluorescence spectrometer was employed for measurements. ROS neutralization was conducted with the same as previously described treatment [29,30,31,32,33,34,35].

Superoxide radical anion (O_2_^.^^−^): (a) Material-Ab (4 µg mL^−1^) was treated with A431 cells, after which it was subjected to 3 h of incubation at 37 °C in darkness. Subsequently, the mixture was exposed to TPE photoexcitation (2816 nJ pixel^−1^, 500 scans; Ex: 800 nm) and finally mixed with 2,3-bis (2-methoxy-4-nitro-5-sulfophenyl)- 2H-tetrazolium-5-carboxanilide (XTT) (0.45 mM; Sigma-Aldrich, St. Louis, MO, USA). The purpose of this material was that it interacted with O_2_**^.^**^−^ and produced XTT-formazan, resulting in strong absorption (470 nm in wavelength). A UV-vis spectrometer was employed to monitor this absorption. For ROS neutralization, the mixture was mixed with 30 ppm of antioxidant α-tocopherol/methyl linoleate (Sigma-Aldrich, St. Louis, MO, USA) in darkness and exposed to TPE photoexcitation with the same treatment. (b) Material-Ab (4 µg mL^−1^) was treated with A431 cells, after which it was subjected to 3 h of incubation at 37 °C in darkness. Subsequently, the mixture was exposed to TPE photoexcitation (2816 nJ pixel^−1^, 500 scans; Ex: 800 nm) and finally mixed with 50 mM bicarbonate buffer (pH 8.60) and glutathione (γ-L-glutamyl-L-cysteinyl-glycine, GSH) (Sigma-Aldrich, St. Louis, MO, USA)/0.80 mM bicarbonate buffer (the Ellman’s assay for O_2_**^.^**^−^ detection). Subsequently, the following experiments were conducted according to the procedure in a previous study. Loss of GSH (%) was calculated as the difference in absorbance between the sample and negative control divided by the absorbance of the negative control. The signal of the generated O_2_**^.^**^−^ was obtained as described in the previous calculation [29,30,31,32,33,34,35]. Data are presented as mean ± SD (*n* = 6).

### 3.13. Singlet Oxygen Quantum Yield (ψ_Δ_) Measurement 

From previous study, ψ_Δ_ can be obtained. ψ_Δ_ measurements were conducted in D_2_O at 355 nm, using *meso*-tetra(4-sulfonatophenyl) porphine dihydrochloride (Sigma-Aldrich, St. Louis, MO, USA) as a reference (ψ_Δ_= 0.64) [36,52].

### 3.14. Uptake Assay

A431 cells were incubated with 4 µg mL^−1^ material-Ab_EGFR_, respectively. The absorbance of a quantity of 4 µg mL^−1^ material-Ab was recorded by UV-vis spectroscopy. The material-Ab_EGFR_ was mixed with A431 cells at 37 °C from 0 h to 8 h, respectively. Then, the mixture was centrifuged (1200 rpm) to remove excess materials, keep the supernatant, and measure the absorbance of the supernatant. The difference between the absorbance values of the collected supernatant and the original materials was estimated, thus resulting in the percentage of uptake at each time point. Data are presented as mean ± SD (*n* = 6).

### 3.15. TPI

A431 cells (5 × 10^3^) were seeded into the 96-well culture dish plates for overnight of incubation in the dark at 37 °C with 5% CO_2_ in air. The materials were mixed into the culture medium (delivered dose of material: 4 µg mL^−1^) for 2.5 h of interaction in the dark at 37 °C to complete the antibody–antigen interaction. The nonspecific binding can be washed out after removal and replacement of the medium for 3 to 5 times. The cells were embedded in a collagen matrix to mimic the 3D epithelium tissue. Further, the TPI of material-treated-A431 cells were observed at 22 °C using a nonlinear femtosecond laser microscopy optical system under TPE.

Furthermore, there is no batch to batch variation for the materials in terms of two-photon properties, and two-photon contrast agents. Different optical system has different detection depth. Due to the detection efficiency and the objective we used, the maximal z depth that can be observed by this laser optical system is approximately 110 μm. However, 105 μm in the work can show the optimal resolution in the mimic 3D biological specimens.

## 4. Conclusions

In this study, we successfully prepared amino-N-GQDs conjugated with polymers containing N (PEI) and S atoms (PSS) to form amino-N-GQD-based nanomaterial polymers, which exhibited a high QY and generated no ROS. The amino-N-GQD polymers also exhibited superior two-photon stability and characteristics among GQD-based nanomaterial polymers, indicating that these polymers can be effective two-photon contrast agents in bioimaging. When the Ab_EGFR_ antibody was employed for labeling to increase specificity in TPI, amino-N-GQD-polymer-Ab_EGFR_-treated cells exhibited high brightness, signal-to-noise ratios, and intensity at an ultralow energy of 16.9 nJ pixel^−1^ under 30 scans and a TPE wavelength of 800 nm (total effective illumination time, ~0.16 s; scan time, 4.53 ms; and scan area, 200 μm × 200 μm). The corresponding imaging depth was 105 μm. These results indicate that the TPE power required to achieve a suitable TPL intensity for amino-N-GQD-polymer-Ab_EGFR_-treated cells is 71 times lower than that required to achieve the same TPAF intensity (TPE power of 1322.8 nJ pixel^−1^ for 500 scans and total effective illumination time of approximately 2.27 s). Thus, cellular photodamage can be minimized. Consequently, amino-N-GQD-polymers are suitable for noninvasive 3D bioimaging.

## Figures and Tables

**Table 1:** TPE cross section of nanomaterials at an excitation wavelength of 800 nm ^a^.

Reference	Integrated Emission Intensity (Counts)		Action Cross-Section (ησ)
Rhodamine B	287.8		155.6
Sample	Integrated emission intensity (counts)	Relative quantum yield (η)	Absolute cross-section (σ)
amino-N-GQD(6.2%)-polymers	78075	0.69	61176
amino-N-GQD(4.9%)-polymers	66405	0.60	59837

^a^ Rhodamine B was selected as the standard reference for the cross section, and the relevant calculations are presented in the Section 3.9 of Materials and Methods.

**Table 2:** Lifetime data and parameters generated using a time-correlated single-photon counting technique involving a triple-exponential fitting function for monitoring the emission at a wavelength of 800 nm under TPE. Material delivered dose: 4 μg mL^−1^ in ddH_2_O.

3 exp Sitting Model: (a0 * exp(a1x) + a2 *exp(a3x) + a4 *exp(a5x) + a6) a1 a3 a5		Lifetime1, Lifetime2, Lifetime3	Average Lifetime (ns)
	−6.57 ± 0.20, −1.15 ± 0.03, −0.27 ± 0.01	0.15 ± 0.01, 0.87 ± 0.02, 3.7 ± 0.16	0.97 ± 0.20
amino-N-GQD			
(6.2%)-polymers			

	−5.92 ± 0.35, −1.08 ± 0.04, −0.28 ± 0.03	0.17 ± 0.01, 0.93 ± 0.04, 3.60 ± 0.37	1.11 ± 0.04
amino-N-GQD			
(4.9%)-polymers			


**Table 3:** Amount of ROS [29,30,31,32,33,34,35] generated, as observed by conducting TPE (2816 nJ pixel^−1^, 500 scans; Ex: 800 nm) on material-Ab-treated-A431 cancer cells. The delivered dose of the material was 4 μg mL^−1^, and the data are presented as means ± SDs (*n* = 6).

		**^1^** **O_2_ (by SOSG)^c^**				
Negative	ROS	Positive	amino-N-	ROS	amino-N-GQD(4.9%)-	ROS
control^ac^	neutralization^abc^	control^cd^	GQD(6.2%)-polymers	neutralization^bc^	polymers	neutralization^bc^
222 ± 10	218 ± 14	2374 ± 118	227 ± 21	220 ± 19	223 ± 16	218 ± 11
		**^1^** **O_2_ (by *t*-MVP)^e^**				
Negative	ROS	Positive	amino-N-	ROS	amino-N-GQD(4.9%)-	ROS
control^ae^	neutralization^abe^	control^de^	GQD(6.2%)-polymers	neutralization^bc^	polymers	neutralization^be^
337 ± 24	333 ± 15	8962 ± 201	341 ± 26	334 ± 22	343 ± 25	337 ± 20
		**O_2_** **˙** **^-^** **(by XTT)^f^**				
Negative	ROS	Positive	amino-N-	ROS	amino-N-GQD(4.9%)-	ROS
control^af^	neutralization^abf^	control^df^	GQD(6.2%)-polymers	neutralization^bf^	polymers	neutralization^bf^
0	0	1.65 ± 0.12	0.06 ± 0.03	0.04 ± 0.01	0.04 ± 0.02	0.03 ± 0.01
		**O_2_** **˙** **^-^** **(by GSH)^g^**				
Negative	ROS	Positive	amino-N-	ROS	amino-N-GQD(4.9%)-	ROS
control^ag^	neutralization^abg^	control^dg^	GQD(6.2%)-polymers	neutralization^bg^	polymers	neutralization^bg^
0	0	94.9 ± 3.5%	0.4 ± 0.1%	0.3 ± 0.1%	0.5 ± 0.2%	0.3 ± 0.1%

^a^ Negative control: only reagent and laser irradiation were used without any material (0 µg mL^−1^). ^b^ ROS neutralization: this process involved nanomaterial treatments, laser irradiation, and treatment with 30 ppm of antioxidant α-tocopherol/methyl linoleate. ^c^ The SOSG reagent (Ex/Em: 488/525 nm) has a suitable specific reactivity for generating fluorescence, which was recorded using a spectrophotometer. ^d^ Positive control: treatment with 50 μM *tert*-butyl hydroperoxide and laser irradiation were performed. ^e^ *t*-MVP (Ex/Em: 352/465 nm) reacted with ^1^O_2_ and formed a dioxetane intermediate, which generated fluorescence upon decomposition to 1-pyrenecarboxaldehyde. This process was monitored using a spectrophotometer. ^f^ XTT interacted with O_2_**^.^**^−^ and produced XTT-formazan, which generated strong absorption (wavelength: 470 nm). ^g^ GSH containing a thiol-tripeptide can prevent damage to cellular or bacterial components caused by oxidation stress. The thiol group from GSH was oxidized to disulfide; thus, GSH was converted into glutathione disulfide. GSH oxidation was used to determine the generated O_2_**^.^**^−^. Loss of GSH (%) = (difference between the absorbance of the sample and negative control/absorbance of negative control) × 100%.

## Supplementary Materials

The following are available online at https://www.mdpi.com/1422-0067/21/8/2939/s1.
