# Supplementary material for: Amino-Functionalized Nitrogen-Doped Graphene-Quantum-Dot-Based Nanomaterials with Nitrogen and Amino-Functionalized Group Content Dependence for Highly Efficient Two-Photon Bioimaging
Source: Int J Mol Sci. 2020 Apr 22;21(8):2939. doi: 10.3390/ijms21082939 (PMC7215431; doi:10.3390/ijms21082939)
Supplement: Supplementary file 1 [file ijms-21-02939-s001.pdf]

Supporting Information

Amino-Functionalized Nitrogen-doped Graphene-Quantum-Dot-based Nanomaterials with Nitrogen and Amino-Functionalized Group Content Dependence for Highly Efficient Two-Photon Bioimaging

Wen-Shuo Kuo[1,2], Chia-Yuan Chang[3], Keng-Shiang Huang[4], Jui-Chang Liu[2,5], Yu-Ting Shao[2,6], Chih-Hui Yang[7,8,9*], and Ping-Ching Wu[10*]*

[1]School of Chemistry and Materials Science, Nanjing University of Information Science and Technology, Nanjing 210044, Jiangsu, China

[2]Allergy & Clinical Immunology Research Center, National Cheng Kung University Hospital, College of Medicine, National Cheng Kung University, Tainan 701, Taiwan (R.O.C.)

[3]Department of Mechanical Engineering, National Cheng Kung University, Tainan 701, Taiwan (R.O.C.)

[4]The School of Chinese Medicine for Post-Baccalaureate, I-Shou University, Kaohsiung 840, Taiwan (R.O.C.)

[5]Department of Biochemistry and Molecular Biology, National Cheng Kung University Hospital, College of Medicine, National Cheng Kung University, Tainan 701, Taiwan (R.O.C.)

[6]Department of Microbiology & Immunology, National Cheng Kung University Hospital, College of Medicine, National Cheng Kung University, Tainan 701, Taiwan (R.O.C.)

[7]Department of Biological Science and Technology, I-Shou University, Kaohsiung 840, Taiwan (R.O.C.)

[8]Pharmacy Department of E-Da Hospital, Kaohsiung 824, Taiwan (R.O.C.)

[9]Taiwan Instrument Research Institute, National Applied Research Laboratories, Hsinchu City 300, Taiwan (R.O.C.)

[10]Department of Biomedical Engineering, National Cheng Kung University, Tainan 701, Taiwan (R.O.C.)

*To whom correspondence should be addressed. E-mail: wskuo88@gmail.com (W.-S. K.); chyang@isu.edu.tw (C.-H. Y.); wbcxyz@bme.ncku.edu.tw (P.-C. W.)

Table S1. Quantitative size distribution. Average diameters of the materials (in ddH_2O) determined by dynamic light scattering [mean lateral size of amino-N-GQD (6.2%)-polymers and amino-N-GQD (4.9%)-polymers: 7.4 ± 0.4 nm and 7.3 ± 0.5 nm, respectively].

amino-N-GQD(6.2%)-polymers	Size (nm)	3.5	4.5	5.5	6.5	7.5	8.5	9.5	10.5
	Fraction (%)	0	3	11	36	35	10	5	0
amino-N-GQD(4.9%)-polymers	Size (nm)	3.5	4.5	5.5	6.5	7.5	8.5	9.5	10.5
	Fraction (%)	0	2	14	33	38	10	3	0

Raman spectroscopy was used to monitor the inelastic phonon scattering caused by the vibration of chemical bonds [1] which enables to calculate the size of the sp^2 domain in the amino-N-GQD and amino-N-GQD-polymers, respectively. If the mean size of the GQD-based nanomaterials > 3 nm, follow the Eq. (1) [2,3],

$$L_a \text{ (nm)} = (2.4 \times 10^{-10})\, \lambda_{laser}^4\, (I_D/I_G)^{-1} \quad (1)$$

where L_a (nm) is the mean size of the sp^2 domain; λ_{Laser} is the excitation wavelength (nm); I means the intensity for the D band and G band, respectively.

However, if the mean size of the GQD-based nanomaterials < 3 nm, follow the Eq. (2) [4].

$$L_D = 0.54\, E_L^4\, (I_D/I_G) \quad (2)$$

where is that in small-size graphene sheets with point-like defects containing sp^3-bonded carbon atoms, the mean distance between defects, L_D (nm), is generally used to represent the size of the sp^2domains $L_{D,}$ (nm); E_{Laser} is the excitation laser energy (eV) used in Raman experiments.

The Raman spectra were obtained with a 532 nm laser and were decomposed into the D band and G band by the Lorentzian function. According to the calculations based on the Eqs. (9-10), the diameter of the amino-N-GQD (6.2%), amino-N-GQD (4.9%), amino-N-GQD (6.2%)-polymers and amino-N-GQD (4.9%)-polymers was 6.9 nm (compared to 7.2 ± 0.5 nm of that determined by HRTEM), 6.8 nm (compared to 7.1 ± 0.7 nm of that determined by HRTEM), 8.0 nm and 7.8 nm. The enlarged diameter of the nanomaterial-polymers was due to the conjugation of polymers.

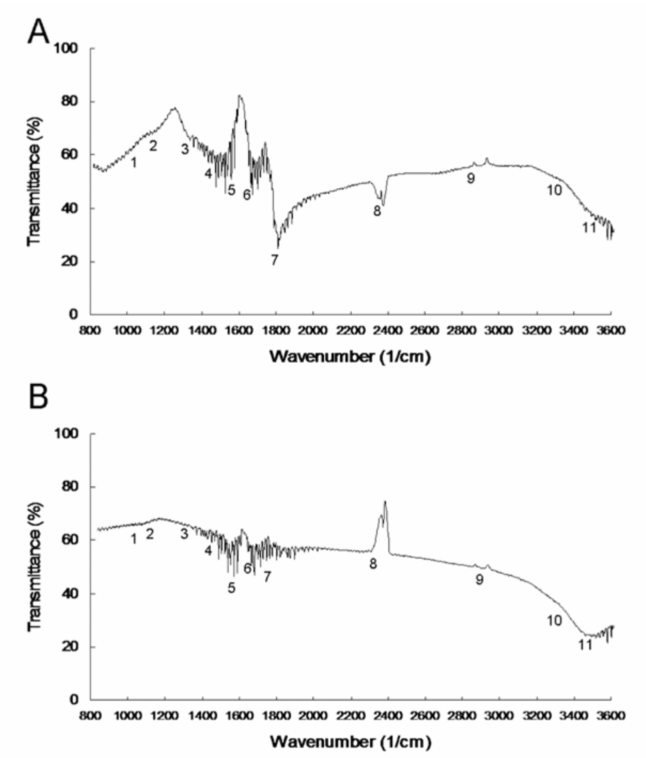

Figure S1. FTIR spectra of (**A**) amino-N-GQD (6.2%)-polymers and (**B**) amino-N-GQD (4.9%)-polymers, respectively. (**A**) The results showed characteristic bands of amino-N-GQDs (6.2%)-polymers forC—N stretching 1020 and 1163 cm^{-1} (bands 1 and 2), for C—H bending 1319 cm^{-1} (band 3), for N—H bending 1426 cm^{-1} (band 4), for N—H bending and scissor 1584, 1638 and 1799 cm^{-1} (bands 5, 6 and 7), for N—H stretching 2357 cm^{-1} (band 8), for C—H stretching 2834 cm^{-1} (band 9), for broad N—H stretching 3277 cm^{-1} (band 10), and N—H vibration about 3440 cm^{-1} (band 11), respectively. (**B**) On the other hand, the characteristic bands of amino-N-GQD (4.9%)-polymers were C—N stretching 1036 and 1149 cm^{-1} (bands 1 and 2), for C—H bending 1310 cm^{-1} (band 3), for N—H bending 1417 cm^{-1} (band 4), for N—H bending and scissor 1580, 1643 and 1781 cm^{-1} (bands 5, 6 and 7), for N—H stretching 2340 cm^{-1} (band 8), for C—H stretching 2855 cm^{-1} (band 9), for broad N—H stretching 3277 cm^{-1} (band 10), and N—H vibration about 3429 cm^{-1} (band 11), respectively.

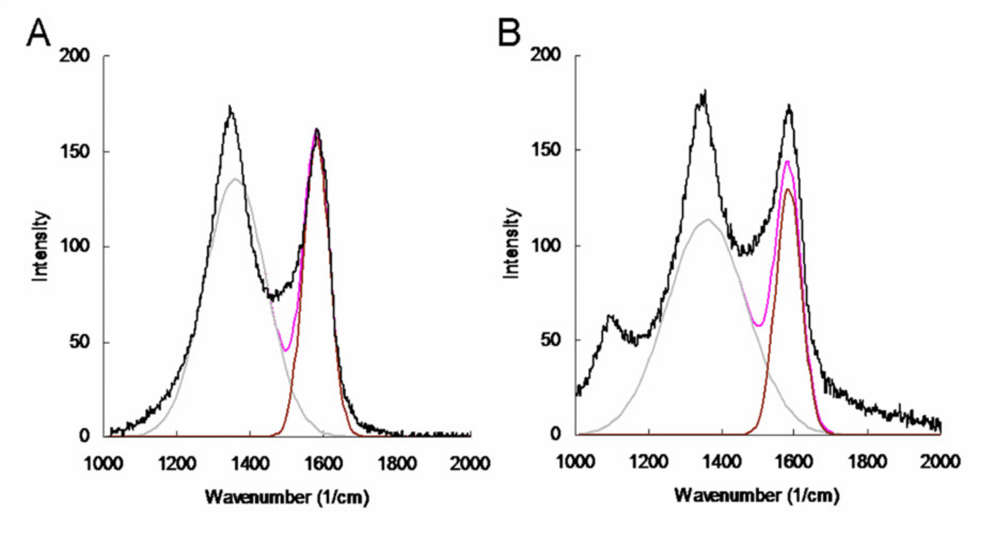

Figure S2. Raman spectra of (**A**) amino-N-GQD (6.2%)-polymers (D band: 1357 cm^{-1}; G band: 1588 cm^{-1}); I_D/I_G integrated intensity ratio of the D and G band: 0.96) and (**B**) amino-N-GQD (4.9%)-polymers (D band: 1355 cm^{-1}; G band: 1586 cm^{-1}); I_D/I_G integrated intensity ratio of the D and G band: 0.99), respectively. Probably because PSS and PEI are electron-donor molecules that cause high-frequency, tangential, vibrational modes of the carbon molecules in the N-GQD-polymers to shift to lower frequencies [5].

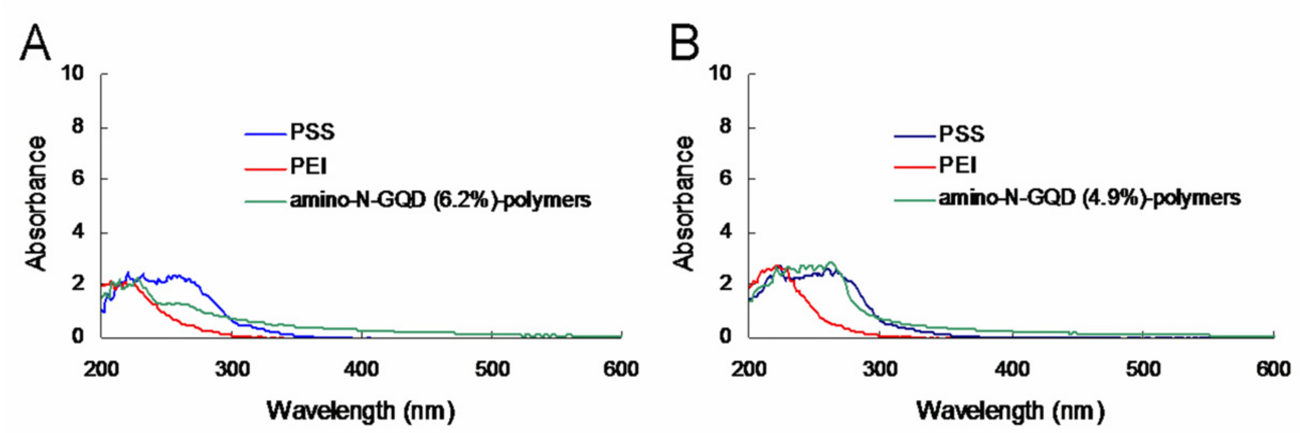

Figure S3. UV-Vis spectra of (**A**) amino-N-GQD (6.2%)-polymers (peaked approximately 232, 263 and 325nm) and (**B**) amino-N-GQD (4.9%)-polymers (peaked approximately 230, 264 and 326 nm), respectively. The broadly characteristic peaks of PSS were shown approximately at 228 nm and 260 nm, and that of PEI was approximately at 220 nm.

Table S2. Amount of ROS [6-12] generated by conducting a TPE (2816 nJ pixel^{-1}, 500 scans; Ex: 800 nm) to material (4 μg mL^{-1}) in ddH_2O was monitored. Data are means ± SD (n=6).

$^{1}O_2$ (by SOSG)[c]

Negative control[ac]	ROS neutralization[abc]	Positive control[cd]	amino-N-GQD(6.2%)-polymers	ROS neutralization[bc]	amino-N-GQD(4.9%)-polymers	ROS neutralization[bc]
224±11	220±13	2361±115	238±24	222±16	235±19	227±14

$^{1}O_2$ (by *t*-MVP)[e]

Negative control[ae]	ROS neutralization[abe]	Positive control[de]	amino-N-GQD(6.2%)-polymers	ROS neutralization[be]	amino-N-GQD(4.9%)-polymers	ROS neutralization[be]
329±22	323±20	8856±197	336±23	331±18	333±21	327±15

$O_2^{\bullet -}$ (by XTT)[f]

Negative control[af]	ROS neutralization[abf]	Positive Control[df]	amino-N-GQD(6.2%)-polymers	ROS neutralization[bf]	amino-N-GQD(4.9%)-polymers	ROS neutralization[bf]
0	0	1.70±0.13	0.05±0.02	0.03±0.01	0.06±0.03	0.02±0.01

$O_2^{\bullet -}$ (by GSH)[g]

Negative control[ag]	ROS neutralization[abg]	Positive Control[dg]	amino-N-GQD(6.2%)-polymers	ROS neutralization[bg]	amino-N-GQD(4.9%)-polymers	ROS neutralization[bg]
0	0	93.5±4.1%	0.6±0.2%	0.2±0.1%	0.4±0.2%	0.2±0.1%

$O_2^{\bullet -}$ (by GSH)[g]

Negative control[ag]	ROS neutralization[abg]	Positive control[dg]	amino-N-GQD(6.2%)-polymers	ROS neutralization[bg]	amino-N-GQD(4.9%)-polymers	ROS neutralization[bg]
0	0	94.9±3.5%	0.4±0.1%	0.3±0.1%	0.5±0.2%	0.3±0.1%

[a]Negative control: only using reagent and laser irradiation without using any material (0 μg mL^{-1}).

[b]ROS neutralization: includes nanomaterial treatments, the laser irradiation and 30 ppm of antioxidant α-tocopherol/methyl linoleate.

[c]The SOSG reagent (Ex/Em: 488/525 nm) has a specific reactivity to generate fluorescence that is recorded using a spectrophotometer.

[d]Positive control: treatment of 50 μM *tert*-butyl hydroperoxide and laser irradiation.

[e]*t*-MVP (Ex/Em: 352/465 nm) can react with $^1O_2$, and form a dioxetane intermediate that generates fluorescence upon decomposition to 1-pyrenecarboxaldehyde. This process is monitored using a spectrophotometer.

[f]XTT can interact with $O_2^{\cdot-}$ and produce XTT-formazan that generates strong absorption (wavelength: 470 nm).

[g]GSH containing a thiol-tripeptide can prevent damages to cellular or bacterial components caused by stress of oxidation. The thiol group from GSH can be oxidized to the disulfide bond, thus converting GSH to glutathione disulfide. GSH oxidation was used to determine the generated $O_2^{\cdot-}$. Loss of GSH (%) = (difference between the absorbance of the sample and negative control / absorbance of negative control) × 100 %.

Table S3. Amount of ROS [6-12] generated by conducting a TPE (2816 nJ pixel^{-1}, 500 scans; Ex: 800 nm) to material (4 μg mL^{-1})-treated-A431 cancer cells was monitored. Data are means ± SD (n=6).

$^1O_2$ (by SOSG)[c]						
Negative control[ac]	ROS neutralization[abc]	Positive control[cd]	amino-N-GQD(6.2%)-polymers	ROS neutralization[bc]	amino-N-GQD(4.9%)-polymers	ROS neutralization[bc]
225±14	219±11	2397±125	235±21	223±17	232±20	224±13

$^1O_2$ (by *t*-MVP)[e]						
Negative control[ae]	ROS neutralization[abe]	Positive Control[de]	amino-N-GQD(6.2%)-polymers	ROS neutralization[be]	amino-N-GQD(4.9%)-polymers	ROS neutralization[be]
335±26	324±14	8952±188	341±25	332±21	328±16	324±12

$O_2 \bullet^-$ (by XTT)[f]						
Negative control[af]	ROS neutralization[abf]	Positive Control[df]	amino-N-GQD(6.2%)-polymers	ROS neutralization[bf]	amino-N-GQD(4.9%)-polymers	ROS neutralization[bf]
0	0	1.67±0.10	0.04±0.02	0.02±0.01	0.05±0.02	0.02±0.01

$O_2 \bullet^-$ (by GSH)[g]						
Negative control[ag]	ROS neutralization[ab]	Positive Control[dg]	amino-N-GQD(6.2%)-polymers	ROS neutralization[bg]	amino-N-GQD(4.9%)-polymers	ROS neutralization[bg]
0	0	94.3±3.8%	0.5±0.3%	0.2±0.1%	0.5±0.2%	0.2±0.1%

[a]Negative control: only using reagent and laser irradiation without using any material (0 μg mL^{-1}).

[b]ROS neutralization: includes nanomaterial treatments, the laser irradiation and 30 ppm of antioxidant α-tocopherol/methyl linoleate.

[c]The SOSG reagent (Ex/Em: 488/525 nm) has a specific reactivity to generate fluorescence that is recorded using a spectrophotometer.

[d]Positive control: treatment of 50 μM *tert*-butyl hydroperoxide and laser irradiation.

[e]*t*-MVP (Ex/Em: 352/465 nm) can react with $^1O_2$, and form a dioxetane intermediate that generates fluorescence upon decomposition to 1-pyrenecarboxaldehyde. This process is monitored using a spectrophotometer.

[f]XTT can interact with $O_2^{\cdot -}$ and produce XTT-formazan that generates strong absorption (wavelength: 470 nm).

[g]GSH containing a thiol-tripeptide can prevent damages to cellular or bacterial

components caused by stress of oxidation. The thiol group from GSH can be oxidized to the disulfide bond, thus converting GSH to glutathione disulfide. GSH oxidation was used to determine the generated $O_2^{\cdot-}$. Loss of GSH (%) = (difference between the absorbance of the sample and negative control / absorbance of negative control) × 100 %.

Table S4. Stability of nanomaterials in physiological environments was determined by dynamic light scattering.

	Mean lateral size (nm)		
	ddH_2O	PBS	culture medium for A431 cells
amino-N-GQD(6.2%)-polymers	7.4 ± 0.4	7.4 ± 0.3	7.5 ± 0.4
amino-N-GQD(4.9%)-polymers	7.3 ± 0.5	7.2 ± 0.4	7.3 ± 0.6

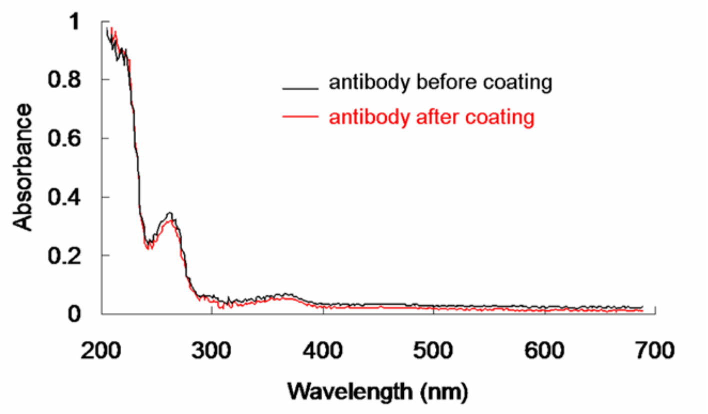

Figure S4. The absorbance of a quantity of antibody before/after coating, and spectra were recorded by UV-vis spectroscopy, respectively (Abs: approximately 270 nm and 394 nm).

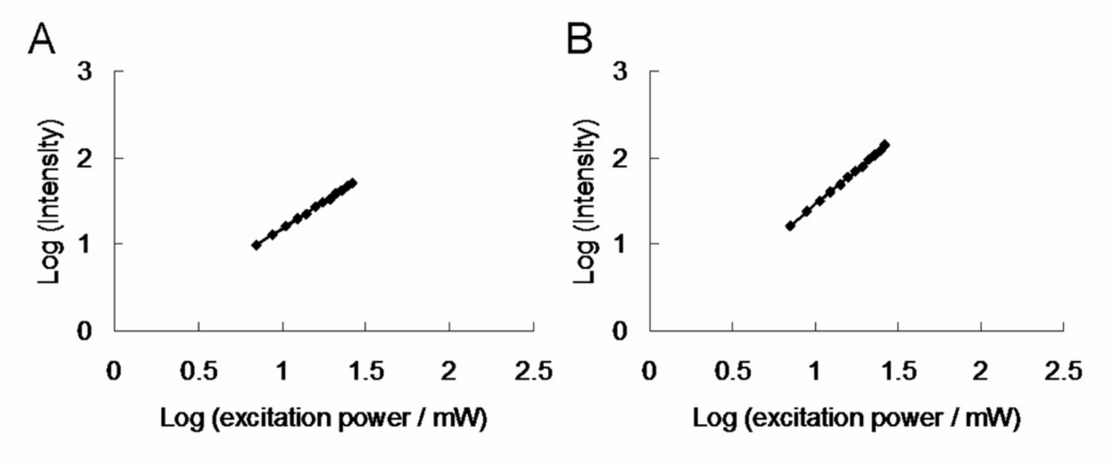

Figure S5. Logarithmic plat of TPL intensity as a function of TPE from 704 nJ pixel^{-1} to 2816 nJ pixel^{-1}. (**A**) Fluorescein (in 0.1M NaOH, pH11) with a slope of 1.98 and (**B**) rhodamine B (in methanol) with a slope of 2.01.R^2>0.999. Excitation wavelength: 800 nm

Table S5. Two-photon action cross sections of fluorescein (in 0.1M NaOH, pH 11) and rhodamine B (in methanol). Excitation wavelength: 800 nm.

	Fluorescein (in ddH_2O, pH=11)	Rhodamine B (in methanol)
Excitation wavelength at 800 nm action cross section, $\eta\sigma_2$ (GM, 10^{-50} cm^4 s/photon)	37.2	155.6

Calculation of radiative and non-radiative decay rates

Upon the absorption of a photon, one of the weakly bound electrons of the fluorescent molecule—a fluorophore—is promoted to a higher energy level. The fluorophore is then in an excited state, A^*. This state is metastable; therefore the fluorophore will return to its stable ground state, A. It can do so either radiatively by emitting a fluorescence photon hv,

$$A^* \ -> \ A + hv$$

or nonradiatively by dissipating the excited state energy as heat

$$A^* \ -> \ A + \text{heat}$$

The depopulation of the excited state depends on the de-excitation pathways available. Fluorescence is the radiative deactivation of the lowest vibrational energy level of the first electronically excited singlet state, S_1, back to the electronic ground state, S_0. The singlet states are the energy levels that can be populated by the weakly bound electron without a spin flip. The absorption and emission processes are illustrated by an energy level diagram named after Aleksander Jablonski.

The fluorescence lifetime, τ, is the average time a fluorophore remains in the electronically excited state S_1 after excitation. τ is defined as the inverse of the sum of the rate parameters for all excited state depopulation processes: Eq. (8), where the nonradiative rate constant k is the sum of the rate constant for internal conversion, k_{ic}, and the rate constant for intersystem crossing to the triplet state, k_{isc}, such that $k= k_{ic},+ k_{isc}$. Fluorescence emission always occurs from the lowest vibrational level of S_1, a rule known as Kasha's rule, indicating that the fluorophore has no memory of its excitation pathway, for example, OPE and TPE yield the same fluorescence spectrum, QY and lifetime.

Cell culture in 3D collagen matrix

A431 cancer cells were cultured in EMEM (EBSS) + 2mM Glutamine + 1% Non Essential Amino Acids + 10% Fetal Bovine Serum at 37℃ under 5% CO_2 in air. Type I collagen (BD, Franklin Lakes, NJ, USA) with original concentration of 4 mg mL^{-1} was diluted with 10X PBS and water to achieve final concentration of 1X PBS and 2 mg mL^{-1} collagen. NaOH was added to neutralize collagen solution before mixing with cells. Cells in serum-free EBSS were mixed with collagen solution. The final concentration of $5 * 10^4$ cells mL^{-1} cell-collagen mixture was added to chambered coverglass (Thermo Scientific, Waltham, MA, USA). Collagen was polymerized at 20°C for 1 hr, and then at 37°C for 20 mins. Full medium was applied after polymerization. Medium was changed everyday after collagen polymerization for both collagen-only and cell-collagen matrix to ensure minimum variation caused by evaporation. Matrices were kept at 37°C, 5% CO_2 incubator. Images were taken at 20°C after collagen polymerization.

References

1. Ferrari, A.C.; Basko, D.M. Raman spectroscopy as a versatile tool for studying the properties of graphene. *Nat. Nanotechnol.* **2013**, *8*, 235–246.
2. Tuinstra, F.; Koenig, J.L. Raman spectrum of graphite. *J. Phys. Chem.* **1970**, *53*, 1126–1130.
3. Pimenta, M.A.; Dresselhaus, G.; Dresselhaus, M.S.; Cancado, L.G.; Jorio, A.; Saito R. Studying disorder in graphite-based systems by Raman spectroscopy. *Phys. Chem. Chem. Phys.* **2007**, *9*, 1276–1290.
4. Ferrari, A.C.; Robertson, J. Interpretation of Raman spectra of disordered and amorphous carbon. *Phys. Rev. B* **2000**, *61*, 14095–14107.
5. Rao A.M.; Eklund, P.C.; Banbow, S.; Thess, A.; Smalley, R.E. Diameter-selective Raman scattering from vibrational modes in carbon nanotubes. *Nature* **1997**, *388*, 257–259.
6. Kuo, W.S.; Chang, C.Y.; Chen, H.H.; Hsu, C.L.L.; Wang, J.Y.; Kao, H.F.; Chou, L.C.S.; Chen, Y.C.; Chen, S.J.; Chang, W.T.; Tseng, S.W.; Wu, P.C.; Pu, Y.C. Two-photon photoexcited photodynamic therapy and contrast agent with antimicrobial graphene quantum dots. *ACS Appl. Mater. Interfaces* **2016**, *8*, 30467–30474.
7. Kinen, M.M.; Kamal-Eldin, A.; Lampi, A.M.; Hopia, A. Effects of α- and γ-tocopherols on formation of hydroperoxides and two decomposition products from methyl linoleate. *J. Am. Oil Chem. Soc.* **2000**, *77*, 801–806.
8. Sharma, P.; Jha, A.B.; Dubey, R.S.; Pessarakli, M. Reactive oxygen species, oxidative damage, and antioxidative defense mechanism in plants under stressful conditions. *J. Bot.* **2012**, *2012*, 1–26.
9. Possel, H.; Noack, H.; Augustin, W.; Keilhoff, G.; Wolf, G. An oxidant, *tert*-butyl hydroperoxide (TBHP), to serve as a positive control. *FEBS Lett.* **1997**, *416*, 175–178.
10. Thompson, A.; Lever, J. R.; Canella, K.A.; Miura, K.A.; Posner, G.H.; Seliger, H.H. Chemiluminescence mechanism and quantum yield of synthetic vinylpyrene analogues of benzo[*a*]Pyrene-7,8-Dihydrodiol. *J. Am. Chem. Soc.* **1986**, *108*, 4498–4504.
11. Ellman, G.L. Tissue sulfhydryl gropus. *Arch. Biochem. Biophys.* **1959**, *82*, 70–77.
12. Carmel-Hare, O.; Storz, G. Roles of the glutathione- and thioredoxin-dependent reduction systems in the *Escherichia coli* and *Saccaromyces cerevisiae* responses to oxidative stress. *Annu. Rev. Microbiol.* **2000**, *54*, 439–461.
